# Catalytic effect of (H_2_O)_*n*_ (*n* = 1–3) on the HO_2_ + NH_2_ → NH_3_ + ^3^O_2_ reaction under tropospheric conditions†

**DOI:** 10.1039/c8ra06549g

**Published:** 2018-11-05

**Authors:** Tianlei Zhang, Kai Wang, Zhangyu Qiao, Yongqi Zhang, Lin Geng, Rui Wang, Zhiyin Wang, Caibin Zhao, Linxia Jin

**Affiliations:** Institute of Theoretical and Computational Chemistry, Shaanxi Key Laboratory of Catalysis, School of Chemical & Environment Science, Shaanxi University of Technology Hanzhong Shaanxi 723001 China ztianlei88@l63.com jinlx@snut.edu.cn +86-0916-2641083 +86-0916-2641083

## Abstract

The effects of (H_2_O)_*n*_ (*n* = 1–3) clusters on the HO_2_ + NH_2_ → NH_3_ + ^3^O_2_ reaction have been investigated by employing high-level quantum chemical calculations with M06-2X and CCSD(T) theoretical methods, and canonical variational transition (CVT) state theory with small curvature tunneling (SCT) correction. The calculated results show that two kinds of reaction, HO_2_⋯(H_2_O)_*n*_ (*n* = 1–3) + NH_2_ and H_2_N⋯(H_2_O)_*n*_ (*n* = 1–3) + HO_2_, are involved in the (H_2_O)_*n*_ (*n* = 1–3) catalyzed HO_2_ + NH_2_ → NH_3_ + ^3^O_2_ reaction. Due to the fact that HO_2_⋯(H_2_O)_*n*_ (*n* = 1–3) complexes have much larger stabilization energies and much higher concentrations than the corresponding complexes of H_2_N⋯(H_2_O)_*n*_ (*n* = 1–3), the atmospheric relevance of the former reaction is more obvious with its effective rate constant of about 1–11 orders of magnitude faster than the corresponding latter reaction at 298 K. Meanwhile, due to the effective rate constant of the H_2_O⋯HO_2_ + NH_2_ reaction being respectively larger by 5–6 and 6–7 orders of magnitude than the corresponding reactions of HO_2_⋯(H_2_O)_2_ + NH_2_ and HO_2_⋯(H_2_O)_3_ + NH_2_, the catalytic effect of (H_2_O)_*n*_ (*n* = 1–3) is mainly taken from the contribution of the water monomer. In addition, the enhancement factor 
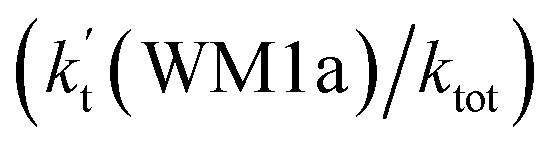
 of the water monomer is 10.06–13.30% within the temperature range of 275–320 K, which shows that at whole calculated temperatures, a positive water effect is obvious under atmospheric conditions.

## Abstract 2

The catalytic effect of (H_2_O)*_n_* (*n* = 1−3) on the HO_2_ + NH_2_ → NH_3_ + ^3^O_2_ is mainly taken from the contribution of a single water vapor.

## Graphical abstract

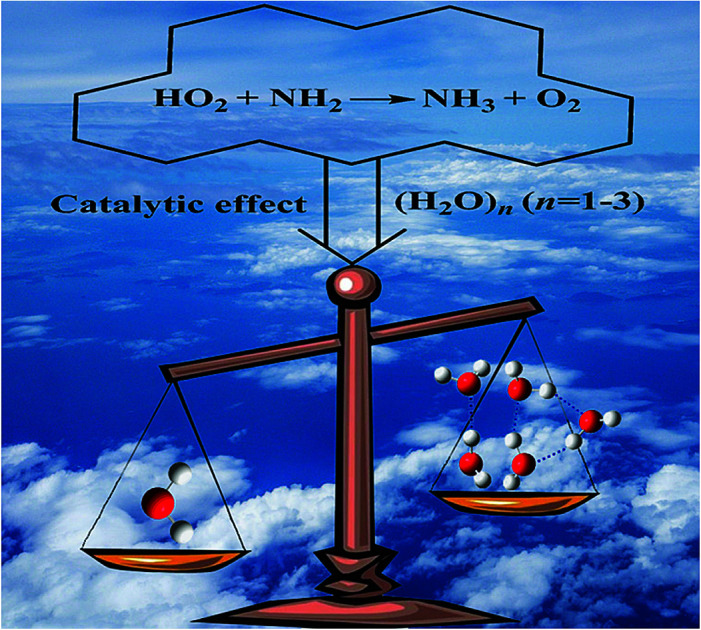

## 1. Introduction

The hydroperoxyl radical (HO_2_) and amidogen radical (NH_2_) are two important reactive radicals in atmospheric processes and combustion chemistry. As one of the most abundant free radicals in the atmosphere, HO_2_ is a very important and reactive intermediate in atmospheric pollutant degradation,^1,2^ photocatalysis,^3^ and molecular oxygen activation on metal surfaces^4,5^ or in enzymes.^6^ Produced from the gas phase oxidation of ammonia (NH_3_) by the hydroxyl radical (eqn (1)), NH_2_ is not only interesting in the combustion of fossil fuels, but is also considered to play an important role in the atmospheric formation and elimination of NO_*x*_ (ref. 7) and in the oxygen isotopic exchange of N_2_O. Meanwhile, NH_2_ reacts with nitric acid can regenerate ammonia (eqn (2)). This reaction and the reaction shown in eqn (1) have been proposed as a potential new catalytic-like cycle which couples the oxidation of ammonia by hydroxyl radicals and the reaction of nitric acid with amidogen radicals in the Earth's atmosphere.^8^ Based on these facts, the kinetics and mechanism of the NH_2_ + HO_2_ reaction (eqn (3)) have been studied experimentally and theoretically.^9–15^

1 NH_3_ + HO → NH_2_ + H_2_O

2 NH_2_ + HNO_3_ → NH_3_ + NO_3_

3 NH_2_ + HO_2_ → NH_3_ + O_2_

On experimental aspect, Sarkisov *et al.*^10^ have measured the gas-phase reaction between NH_2_ and HO_2_ by the analytical technique of the VIS-UV absorption at a total pressure of 133–9.33 × 10^4^ Pa and the rate constant of the reaction (3) has been found to be 7.51 × 10^−11^ cm^3^ per molecule per s at 300 K. Meanwhile, the rate constant of NH_2_ + HO_2_ reaction has been estimated indirectly by flash-photolysis, which is 2.5 × 10^−11^ cm^3^ per molecule per s at 300 K.^9^ Theoretically, Sumathi *et al.*^14^ obtained the singlet potential energy surface of reaction (3) at QCISD(T)/6–311++ G(2*df*,2*pd*)//6–311++G**/MP2 level. In their work, to obtain the ratio of their product formation and to estimate the contribution of different channels over a wide range of temperature, the primary concern is to analyze the competition among the various reaction channels. However, the triplet potential energy surface of reaction (3), especially, the triplet hydrogen abstraction (HA) is not involved, which is not neglected for the HA in many previous reports reaction between radical and HO_2_ radical.^16–30^ Even in some HA reaction, triplet HA is favorable kinetically.^16,18,20,23,26,29,30^ So, both the singlet and triplet HA have been investigated at the CCSD(T)//B3LYP/6–311++G(3*df*,3*pd*) level by Xiang *et al.*^31^ In their work, for the favorable HA, the reaction mechanism on the triplet potential surface to be mainly a barrierless addition of HO_2_ to NH_2_ leading to an intermediate OOH⋯NH_2_ (^3^im1), and then the adduct ^3^im1 goes through an H transfer forming the product of NH_3_ and ^3^O_2_.

These investigations provide meaningful information about the HA of NH_2_ + HO_2_ reaction under atmospheric conditions. Nevertheless, these studies did not take into consideration the influence of water vapors on the reaction. In fact, firstly, water is ubiquitous in the Earth's atmosphere and its monomer can form hydrogen bonded complexes with other abundant radicals changing their photochemical features.^32^ Such as previous investigations showed that in the process of HO_2_ self-reaction, hydrogen bonded complexes HO_2_⋯H_2_O are formed with approximately 30% of the HO_2_ in the atmosphere bonding with water under typical atmospheric conditions.^33^ Another example is that water monomer can bind with NH_2_ radical, forming H_2_O⋯H_2_N, and H_2_N⋯H_2_O complexes.^34^ Secondly, it is also known that water was found to actively participate in the atmospheric reactions of HO_2_ + HO_2_,^25,35^ HO_2_ + HS,^28^ HO_2_ + HO,^26,36^ HO_2_ + SO_2_,^37^ HO_2_ + NO_2_ (ref. 24) and HO_2_ + O_3_(ref. 38 and 39) reactions. Meanwhile, in these processes, water vapor had a catalytic effect by increasing the stability of pre-reactive complexes and reducing the activation energy of transition states. The above facts forecast that it cannot ignore water in modeling the different atmospheric HA reactions. These situations stimulated our interest in modeling the gas-phase reaction of H_2_O⋯HO_2_⋯NH_2_ ternary system, in which the single water molecule serves as a catalyst.

Although atmospheric water molecule implies a significant catalytic effect by monomers, the catalytic effect of water dimers and also water trimers can't be ignored, because their concentrations are up to 9 × 10^14^ and 2.6 × 10^12^ molecules per cm^3^ at 298 K.^40,41^ Moreover, the experimental and theoretical studies have been reported in the literature on the electronic structure of the clusters HO_2_⋯(H_2_O)_*n*_ (*n* = 2–3).^28,39^ Thus, the catalytic effects of (H_2_O)_*n*_ (*n* = 2–3) are worth being investigated further on the HO_2_ + NH_2_ → NH_3_ + ^3^O_2_ reaction.

In the present study, based on the HO_2_ + NH_2_ → NH_3_ + ^3^O_2_ reaction without water molecule, a detailed effects of (H_2_O)_*n*_ (*n* = 1–3) on the HA reaction of HO_2_ + NH_2_ → NH_3_ + ^3^O_2_ have been studied at the CCSD(T)/CBS//M06-2X/6-311+G(3*df*,2*pd*) level of theory, which is organized as follows: firstly, the triplet HA reaction of HO_2_ + NH_2_ → NH_3_ + ^3^O_2_ was investigated to compare with (H_2_O)_*n*_ (*n* = 1–3)-assisted processes. Secondly, the reactions of H_2_O⋯HO_2_ + NH_2_, HO_2_⋯H_2_O + NH_2_, H_2_O⋯H_2_N + HO_2_ and H_2_N⋯H_2_O + HO_2_ with water monomer were evaluated by investigating direct HA process and double hydrogen transfer mechanism. In what follows, direct HA processes of HO_2_⋯(H_2_O)_2_ (water dimer and the whole HO_2_ radical formed a ring by hydrogen bonds) + NH_2_, HO_2_⋯(H_2_O)_2_–I (water dimer and the HO moiety of HO_2_ radical formed a ring by hydrogen bonds) + NH_2_ and H_2_N⋯(H_2_O)_2_ + HO_2_ reactions with (H_2_O)_2_ were also calculated. Then, based on the discussed results of water dimer, the reactions of HO_2_⋯(H_2_O)_3_ + NH_2_ and H_2_N⋯(H_2_O)_3_ + HO_2_ were mainly investigated for the channel of NH_3_ + ^3^O_2_ formations with water trimer. Finally, the effective rate constants of the HA reaction of HO_2_ + NH_2_ → NH_3_ + ^3^O_2_ with (H_2_O)_*n*_ (*n* = 1–3) were calculated to investigate the atmospheric relevance of the effect of (H_2_O)_*n*_ (*n* = 1–3). Overall, this work may lead to a better understanding of the effects of (H_2_O)_*n*_ (*n* = 1–3) on the gas-phase reactions under tropospheric conditions.

## 2. Computational methods

### 2.1 Electronic structure calculation

The electronic structure calculations were performed using Gaussian 09 program package^42^ software. The geometries of all the reactants, intermediates, transition states and products have been optimized using the M06-2X^43–46^ method using the 6-311+G(3*df*,2*pd*) basis set. The corresponding frequencies of the optimized geometries were computed at the same level to confirm the characteristics of the transition states with one imaginary frequency and the stationary points without imaginary frequency. Moreover, the minimum energy path (MEP) was obtained by the intrinsic reaction coordinate (IRC)^47–49^ theory with a gradient step size of 0.01 − 0.05 (amu)^1/2^ bohr to prove that the TS connects to minima along the reaction path. In order to obtain more accurate relative energies, single-point energy calculations for the stationary points were performed at the CCSD(T) method^50^ in conjunction with CBS basis set based on the M06-2X/6-311+G(3*df*,2*pd*)-optimized geometries. The single point energy calculations of CCSD(T)/CBS have been carried out for all the species at CCSD(T) level of theory using aug-*cc*-pVDZ and aug-*cc*-pVTZ basis sets. The energy values obtained at DZ and TZ levels have been used to extrapolate the results to a complete basis set (CBS) limit.^51^

### 2.2 Rate constant calculations

To estimate the effect of (H_2_O)_*n*_ (*n* = 1–3) added, the theoretical rate constants of canonical variational transition (CVT) state theory^52–54^ with small curvature tunneling (SCT) correction^55,56^ for every H-abstraction channels were calculated by employing VKLab program^57^ coupled with the steady state approximation.

In the presence of (H_2_O)_*n*_ (*n* = 1–3), all the processes for the formations of NH_3_ and ^3^O_2_ from the reaction of HO_2_ and NH_2_ involve two major steps as follows.
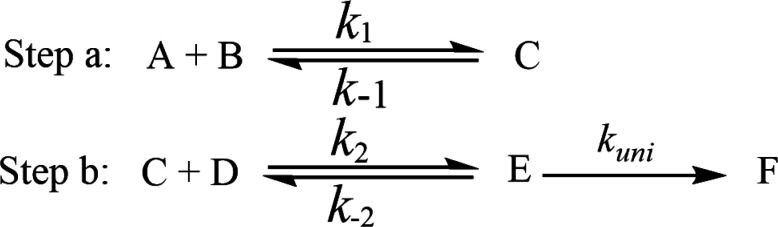


Here, A and B are any two among HO_2_, NH_2_ and (H_2_O)_*n*_ (*n* = 1–3) clusters (water monomer, WM; water dimer, WD; and water trimer, WT), C is the binary complex formed by A and B. D is the remaining third species other than A and B. E is the ternary complex formed by HO_2_, NH_2_ and (H_2_O)_*n*_ (*n* = 1–3). In the step a, A combines with B to from an adduct C, whereas the step b consists of two elementary processes: in the first one, C reacts with D to form E and subsequently E undergoes uni-molecular transformation to produce the formation F *via* the corresponding TS.

Assuming that the intermediate E was in equilibrium with the corresponding reactants (C and D) and was at steady state,^58^ the rate constant for step b can be written as4
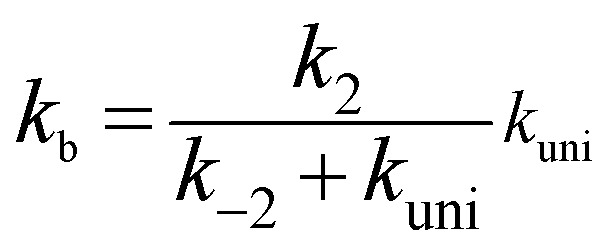


If *k*_uni_ ≪ *k*_−2_, the rate constant of *k*_b_ was rewritten as5
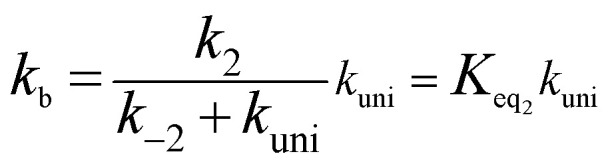


The rate constant *k*_uni_ in eqn (5) has been evaluated by VKLab program^57^ in the framework of the canonical variational transition state theory (CVT).^54^ To include the tunneling effects for motion along the reaction coordinate for the title reactions at the CCSD(T)/CBS//M06-2X/6-311+G(3*df*,2*pd*) level, the small curvature tunneling (SCT)^55^ approximation has been adopted in this study. Besides, *K*_eq_2__ in eqn (5) was given by eqn (6).6
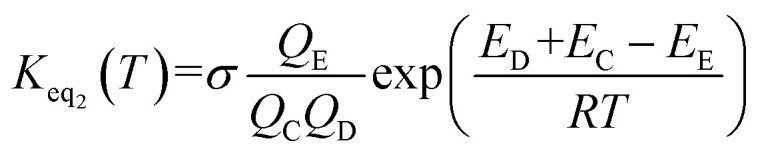


In eqn (6), the various *Q* values denote the partition functions of the intermediate E, reactants C and D, respectively. All partition functions were obtained using the M06-2X/6-311+G(3*df*,2*pd*) method. *E*_D_, *E*_C_ and *E*_E_ stand for the energies of the species of D, C and E, respectively; *σ* is the symmetry factor. In the present work, *k*_b_ has been used to compare the rates between bare reaction and catalyzed reactions.

### 2.3 Effective rate constant calculations

If one incorporates the effect of step a, the resultant rate constant (*k*_t_) can be written as:

7 *k*_t_ = *K*_eq_1__*K*_eq_2__*k*_uni_

where *K*_eq_1__ stands for the equilibrium constant in step a *i.e. K*_eq_1__ = *k*_1_/*k*_−1_, and *K*_eq_1__ was given by eqn (8).8
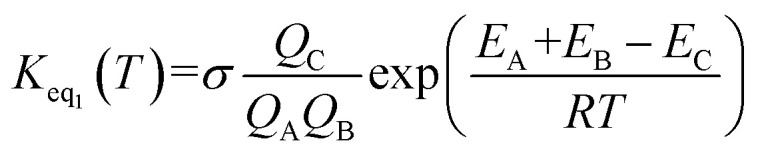


In eqn (8), the various *Q* values denote the partition functions of the complex C, reactants A and B, respectively. *E*_A_, *E*_B_ and *E*_C_ stand for the energies of the species of A, B and C, respectively. From the above, the rate of the reaction (*v*) in the presence of catalysts can be written as:9
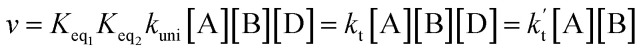

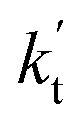
 is the effective rate constant, which could be considered as a measure of the relative efficiencies of the different catalysts under atmospheric conditions, as it includes the concentration as well as rate constant of a particular catalyst.

## 3. Results and discussion

The transition states in each reaction channel were signed by “TS” followed by a number, and intermediates were denoted by “IM” followed by a number. The letters “a”, “b”, and “c” were used to distinguish the transition states and intermediates that were conformers of each other and therefore had the same features; a species in the presence of water monomer, water dimer and water trimer was respectively denoted by a “WM”, “WD”, and “WT” suffix.

### 3.1 Potential energy surfaces and the rate constants for the hydrogen abstraction of HO_2_ + NH_2_ → NH_3_ + ^3^O_2_ reaction

**Fig. 1:**
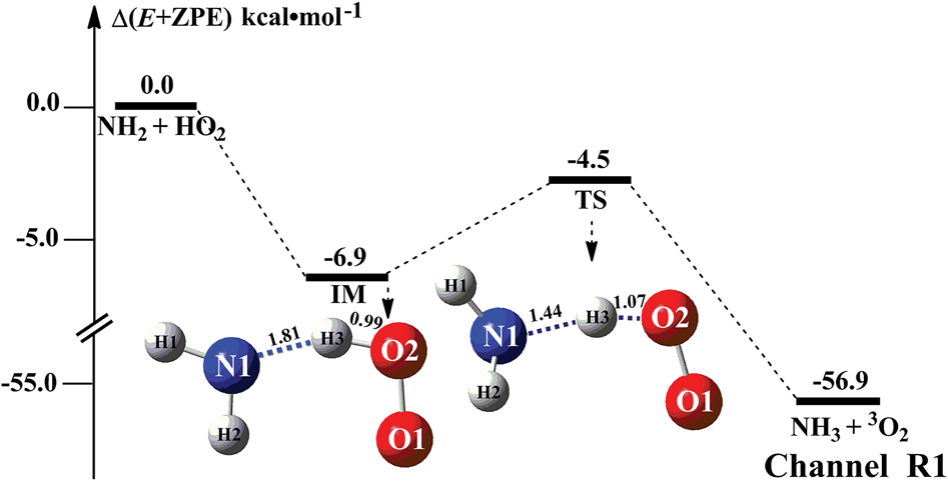
Schematic energy diagram for the HO_2_ + NH_2_ reaction; energies (kcal mol^−1^) at the CCSD(T)/CBS//M06-2X/6-311+G(3*df*,2*pd*)) level of theory.

The HO_2_ + NH_2_ → NH_3_ + ^3^O_2_ reaction was investigated theoretically by Xiang *et al.*^31^ at the CCSD(T)/6–311++G(3*df*,3*pd*)//B3LYP/6–311++G(3*df*,3*pd*) level. In this study, we have reinvestigated that work at the CCSD(T)/CBS//M06-2X/6-311+G(3*df*,2*pd*) level to determine the outcome of this reaction when (H_2_O)_*n*_ (*n* = 1–3) was present. As seen in Fig. 1, regarding the HA reaction of HO_2_ + NH_2_ to produce NH_3_ + ^3^O_2_, only one elementary reaction path was identified (Channel R1). Starting from HO_2_ + NH_2_ reactants, Channel R1 began with the intermediate IM. The binding energy of IM was 6.9 kcal mol^−1^, agree with that (^3^im1, 7.4 kcal mol^−1^) in the literature reported by Xiang *et al.*^31^ For IM, from geometrical point of view, a hydrogen bond shown in Fig. 1 was formed between the H atom of HO_2_ and the N atom of NH_2_ radical (with a computed H⋯N bond distance of 1.81 Å at the M06-2X/6-311+G(3*df*,2*pd*) level of theory). With the N atom of NH_2_ radical attacks the H atom of HO_2_, the intermediate IM in Channel R1 proceeded through a transition state TS with the energy predicted to be at 4.5 kcal mol^−1^ below the initial reactants. The energy of TS is quite different from ^3^ts3 (6.8 kcal mol^−1^ below HO_2_ + NH_2_ reactants) in the literature reported by Xiang *et al.*^31^ This difference is possible due to the fact that the different levels of theory used for the ZPE and single-point energy calculation. Besides, the Δ*H* (298) of HO_2_ + NH_2_ → NH_3_ + ^3^O_2_ reaction was predicted to be −57.0 kcal mol^−1^, compared with the experimental^59^ estimation of −58.5 ± 0.4 kcal mol^−1^. For reaction (1) without water, Table S1† lists its CVT/SCT rate constant. As seen in Table S1,† tunneling slightly increases the rate constant, while the recrossing effects decrease the rate constant. For example, the rate constant is increased by 62% due to tunneling, while the rate constant is decreased to 52% because of recrossing effects at the M06-2X/6-311+G(3*df*,2*pd*) level and at 298 K (Table S1†). It is noted that tunneling and recrossing effects slightly depend on temperature. Tunneling slightly increases with the decrease of temperature, while recrossing effects slightly increase with the increase of temperature as listed in Table S1.† Specifically, the calculated results using M06-2X/aug-*cc*-pVTZ indicate that the rate constant is increased by 32% and 20% due to tunneling, while the rate constant is decreased to 44% and 60% at 275 and 320 K, respectively. So, similar with previous investigations,^60,61^ the tunneling transmission coefficients are very large for the hydrogen atom transfer process. Thus, herein the computed CVT/SCT rate constants have been used to estimate the catalytic effect of (H_2_O)_*n*_ (*n* = 1–3). CVT/SCT rate constant calculations were carried out for Channel R1 at various temperatures. The values are listed in Table S1.† At 300 K, the calculated value of *k*_R_1__ was 2.68 × 10^−11^ cm^3^ per molecule per s_._ The calculated values compare closely with 7.51 × 10^−11^ cm^3^ per molecule per s and 2.5 × 10^−11^ cm^3^ per molecule per s, at 300 K respectively predicted by Sarkisov *et al.*^10^ and Cheskis *et al.*^9^ indicating that the calculations for the HO_2_ + NH_2_ → NH_3_ + ^3^O_2_ reaction without and with (H_2_O)_*n*_ (*n* = 1–3) at the CCSD(T)/CBS//M06-2X/6-311+G(3*df*,2*pd*) level of theory are acceptable.

### 3.2 Geometrical analysis and the concentration calculation for HO_2_⋯(H_2_O)_*n*_ (*n* = 1–3) and H_2_N⋯(H_2_O)_*n*_ (*n* = 1–3) complexes

**Fig. 2:**
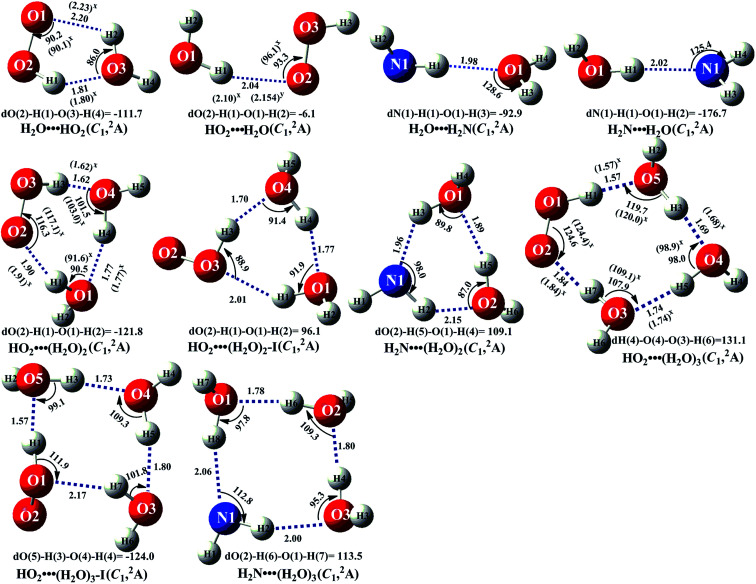
The geometrical structures of the optimized complexes at the M06-2X/6-311+G(3*df*,2*pd*) level of theory (bond length Å, bond angle °).

In the presence of (H_2_O)_*n*_ (*n* = 1–3), both bodies of HO_2_ and NH_2_ can respectively interact with the second body of (H_2_O)_*n*_ (*n* = 1–3) *via* hydrogen bond to form two-body complexes of HO_2_⋯(H_2_O)_*n*_ (*n* = 1–3) and H_2_N⋯(H_2_O)_*n*_ (*n* = 1–3) first in the entrance channels before interacting with the third body of NH_2_ or HO_2_. So, it is very necessary to find the stable configurations of the complexes HO_2_⋯(H_2_O)_*n*_ (*n* = 1–3) and H_2_N⋯(H_2_O)_*n*_ (*n* = 1–3) firstly. In order to find all possible stable configurations of the complexes HO_2_⋯(H_2_O)_*n*_ (*n* = 1–3) and H_2_N⋯(H_2_O)_*n*_ (*n* = 1–3), the global minimum searching of geometric structures were carried out using Tsinghua Global Minimum (TGMin).^62,63^ Then the initial structures for HO_2_⋯(H_2_O)_*n*_ (*n* = 1–3) and H_2_N⋯(H_2_O)_*n*_ (*n* = 1–3) were selected for geometry optimization using the M06-2X/6-31G(*d*) method. The isomer structures within 6.0 kcal mol^−1^ of the global minimum were re-optimized by M06-2X/6-311+G(3*df*,2*pd*) method. The Fig. 2 and S1† show the optimized geometrical reactants of HO_2_⋯(H_2_O)_*n*_ (*n* = 1–3) and H_2_N⋯(H_2_O)_*n*_ (*n* = 1–3), which are in good agreement with available previous results.^64,65^ As seen in Fig. 2 and S1,† as the number of water molecules increases in a given cluster of HO_2_⋯(H_2_O)_*n*_ (*n* = 1–3) and H_2_N⋯(H_2_O)_*n*_ (*n* = 1–3), the number of possible configurations for the cluster increases quickly, and each of the configurations becomes increasingly complicated. Regarding to each type equilibrium structure of HO_2_⋯(H_2_O)_*n*_ (*n* = 1–3) and H_2_N⋯(H_2_O)_*n*_ (*n* = 1–3), herein we only focus on the stable configuration shown in Fig. 2, which has the larger stabilization energy and the higher concentration than its isomers.

As seen in Fig. 2 and Table S2,† consistent with previous reports,^24,25,28,37,66^ five-membered ring complex H_2_O⋯HO_2_ was much more stable than the single hydrogen bond complexes HO_2_⋯H_2_O, H_2_N⋯H_2_O and H_2_O⋯H_2_N with its binding energy larger by 3.6–5.9 kcal mol^−1^ than those of latter ones. The equilibrium constants of these complexes at 298 K are 1.83 × 10^−19^, 3.05 × 10^−22^, 1.34 × 10^−22^ and 8.40 × 10^−21^ cm^3^ per molecule, respectively (Table S3†). Considering typical tropospheric concentrations of 7.73 × 10^17^ molecules per cm^3^ of H_2_O, 3 × 10^8^ molecules per cm^3^ of HO_2_,^67^ and our estimated concentrations of NH_2_ (6.0 × 10^−11^ molecules per cm^3^ seen in ESI†), it is estimated that the atmospheric concentration of the H_2_O⋯HO_2_ complex to be 4.24 × 10^7^ molecules per cm^3^, which is larger by 6.00 × 10^2^ times than that of HO_2_⋯H_2_O. However, in our previous works,^25,26^ the reaction channels of HO_2_⋯H_2_O + HO_2_ and HO_2_⋯H_2_O + HO were not neglected in water-catalyzed HO_2_ + HO_2_ and HO_2_ + HO. So, for water catalyzed HO_2_ + NH_2_ → NH_3_ + ^3^O_2_ reaction, both H_2_O⋯HO_2_ + NH_2_ and HO_2_⋯H_2_O + NH_2_ reactions have been investigated in the following section. Besides these, at 298 K, the concentrations of H_2_N⋯H_2_O (3.90 × 10^−13^ cm^3^ per molecule) and H_2_O⋯H_2_N (6.21 × 10^−15^ cm^3^ per molecule) shown in Table S3† were much lower than those of H_2_O⋯HO_2_ (4.24 × 10^7^ cm^3^ per molecule) and HO_2_⋯H_2_O (7.07 × 10^4^ cm^3^ per molecule). Thus, in water catalyzed HO_2_ + NH_2_ → NH_3_ + ^3^O_2_ reaction, we predict that the atmospheric relevance of H_2_O⋯HO_2_ + NH_2_ and HO_2_⋯H_2_O + NH_2_ reactions will be much more obvious than those of H_2_N⋯H_2_O + HO_2_ and H_2_O⋯H_2_N + HO_2_ reactions. Thus, only H_2_O⋯HO_2_ + NH_2_ and HO_2_⋯H_2_O + NH_2_ reactions have been taken into account in the following section, whereas, for comparison, the potential energy surfaces (PESs) for H_2_N⋯H_2_O + HO_2_ and H_2_O⋯H_2_N + HO_2_ reactions has been displayed in Fig. S5,† and their corresponding rate constants were shown in Table S7.†

For the clusters constituted by HO_2_ (or NH_2_) radical and water dimer, in geometrical point of view, HO_2_⋯(H_2_O)_2_ (water dimer and the whole HO_2_ radical formed a ring by hydrogen bonds) shows seven-membered ring structure, whereas both HO_2_⋯(H_2_O)_2_–I (water dimer and the HO moiety of HO_2_ radical formed a ring by hydrogen bonds) and H_2_N⋯(H_2_O)_2_ involves a six-membered ring. So, the binding energy of HO_2_⋯(H_2_O)_2_ (shown in Table S2†) was 12.6 kcal mol^−1^, which was larger by 3.9–6.4 kcal mol^−1^ than those of HO_2_⋯(H_2_O)_2_–I and H_2_N⋯(H_2_O)_2_ due to smaller ring tension. Similarity, nine-membered ring HO_2_⋯(H_2_O)_3_ (water trimer and the whole HO_2_ radical formed a ring by hydrogen bonds) was larger by 2.0–7.3 kcal mol^−1^ than those of eight-membered ring HO_2_⋯(H_2_O)_3_–I (water trimer and the HO moiety of HO_2_ radical formed a ring by hydrogen bonds) and H_2_N⋯(H_2_O)_3_. From another point of view shown in Table S3,† the concentrations of H_2_N⋯(H_2_O)_2_ (5.13 × 10^−17^ cm^3^ per molecule) and H_2_N⋯(H_2_O)_3_ (1.02 × 10^−19^ cm^3^ per molecule) are also much lower than those of HO_2_⋯(H_2_O)_2_ (5.14 × 10^5^ cm^3^ per molecule) and HO_2_⋯(H_2_O)_3_ (8.02 × 10^3^ cm^3^ per molecule) at 298 K, thus we predict that the atmospheric relevance of H_2_N⋯(H_2_O)_2_ + HO_2_ and H_2_N⋯(H_2_O)_3_ + HO_2_ reactions can be neglected. However, for comparison, the PESs for H_2_N⋯(H_2_O)_2_ + HO_2_ and H_2_N⋯(H_2_O)_3_ + HO_2_ reactions have been displayed in Fig. S6 and S7,† and their corresponding rate constants were shown in Table S7.† Besides these, the concentrations of HO_2_⋯(H_2_O)_2_ and HO_2_⋯(H_2_O)_3_ are respectively larger by 140 and 9 times than the corresponding complexes of HO_2_⋯(H_2_O)_2_–I and HO_2_⋯(H_2_O)_3_–I at 298 K. So, we predict that the catalytic effect of HO_2_⋯(H_2_O)_2_–I + NH_2_ and HO_2_⋯(H_2_O)_3_–I + NH_2_ reactions are less obvious than the corresponding reactions of HO_2_⋯(H_2_O)_2_ + NH_2_ and HO_2_⋯(H_2_O)_3_ + NH_2_.

### 3.3 Potential energy surfaces and the rate constants for HO_2_ + NH_2_ → NH_3_ + ^3^O_2_ reaction with H_2_O

**Fig. 3:**
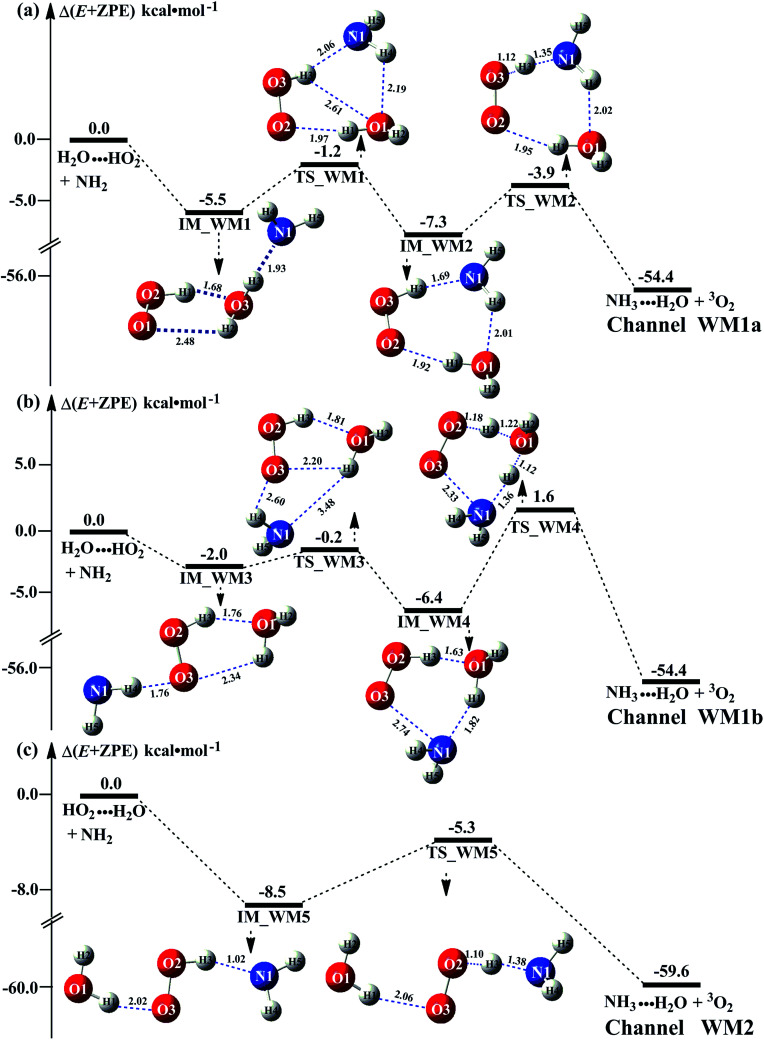
Schematic energy diagram for the water assisted HO_2_ + NH_2_ → NH_3_ + ^3^O_2_ reaction occurring through H_2_O⋯HO_2_ + NH_2_ (a and b), and HO_2_⋯H_2_O + NH_2_ (c) reactions; energies (kcal mol^−1^) at the CCSD(T)/CBS//M06-2X/6-311+G(3*df*,2*pd*) level of theory.

It has been pointed out in Section 3.2 that water-catalyzed the reaction (3) is mainly occurring through H_2_O⋯HO_2_ + NH_2_ and HO_2_⋯H_2_O + NH_2_ reactions. So, Fig. 3 presents the PESs for H_2_O⋯HO_2_ + NH_2_ (Channel WM1) and HO_2_⋯H_2_O + NH_2_ (Channel WM2) reactions along with the local minimum geometries on the corresponding reaction pathways.

Regarding to Channel WM1, by different collisions between H_2_O⋯HO_2_ and NH_2_, two kinds of reaction types have been found, which were labeled as Channel WM1a and Channel WM1b. For Channel WM1a, starting from H_2_O⋯HO_2_ + NH_2_ reactants, hydrogen-bonded complex IM_WM1 was formed by the interaction between N atom of NH_2_ radical and one H atom of the H_2_O moiety in H_2_O⋯HO_2_ complex with a bonding energy of 5.5 kcal mol^−1^. After a flat potential energy surface through TS_WM1, with an energy barrier of 4.3 kcal mol^−1^, the formation of seven-member cyclic complex IM_WM2 was formed with a binding energy of 7.3 kcal mol^−1^ relative to H_2_O⋯HO_2_ + NH_2_ reactants. Following complex IM_WM2, Channel WM1a proceeded through transition state TS_WM2 to produce the product of NH_3_⋯H_2_O and ^3^O_2_ after climbing the barrier height of 3.4 kcal mol^−1^. In the transition state TS_WM2, the seven-membered ring structure was still conserved with the N atom of NH_2_ radical abstracting the H atom of HO_2_ moiety in H_2_O⋯HO_2_ complex.

Similar with Channel WM1a, Channel WM1b also followed stepwise process. In the first step, similar with the ring enlargement from IM_WM1 to IM_WM2 in Channel WM1a, the five-membered ring complex IM_WM3 is rearranged into seven-membered ring complex IM_WM4 through TS_WM3. In geometrical point of view, complex IM_WM4 has similar seven-membered cyclic structure as IM_WM2 with the NH_2_ radical and the water molecule exchanging their positions. The binding energy of IM_WM4 is 6.4 kcal mol^−1^, lowered by 0.9 kcal mol^−1^ than that of IM_WM2. In the second step, differently from transition state TS_WM2 in Channel WM1a that involves a direct HA, transition state TS_WM4 in Channel WM1b contains a double hydrogen transfer mechanism. Consistent with our previous reports,^28^ such mechanism discrepancy between Channels WM1a and WM1b may lead that the energy barrier of the second step in Channel WM1a is 3.4 kcal mol^−1^, which is lower by 4.6 kcal mol^−1^ than that of the second step in Channel WM1b. The reason can be possibly explained in three following aspects: (1) transition state TS_WM2 in the former reaction shows seven-member ring structure, whereas the transition state TS_WM4 in the latter reaction shows six-member ring structure. That is, TS_WM4 has larger ring tension than that of TS_WM2; (2) different from the structures in the process of IM_WM4 → TS_WM4 where the three hydrogen atoms (H2, H4 and H5) are out of plane, the structure in the process of IM_WM2 → TS_WM2 is close to the coplanar structure, which makes the conjugated hydrogen bonding system (H3⋯N1, H4⋯O1 and H1⋯O2) more stable; (3) NBO charge analysis shows that (Fig. S8†), the distance between the negatively NBO charged O3 and N1 atoms was decreases from IM-WM4 (2.70 Å) → TS-WM4 (2.33 Å), resulting in enhanced repulsive interactions, and hence the energy of the system is increased.

As shown in Table 1, it is worth noting that the rate constants both of Channel WM1a (*k*_b_(WM1a)) and Channel WM1b (*k*_b_(WM1b)) are increased with the decrease of temperature. This is because the calculated bimolecular rate constant contains two different components, (see Table S7†)); (1) *K*_eq_ from the first step (bimolecular addition between H_2_O⋯HO_2_ and NH_2_) and (2) *k*_2_ from the second step (IM_WM1 (or IM_WM3) → NH_3_⋯H_2_O + ^3^O_2_). The second step always contributes to the positive activation energy due to the finite positive barrier, while the first step corresponds to negative activation energy as it involves barrierless addition of isolated reactants to form the complex IM_WM1 (or IM_WM3). Consequently, *K*_eq_ always decreases with increase in temperature, whereas *k*_2_ behaves in opposite manner (Table S7†). Whenever *k*_2_ dominates over *K*_eq_ the overall activation energy of the reaction is found to be positive and when *K*_eq_ dominates over *k*_2_ then the overall activation energy of the reaction become negative. For example, for Channel RW1a, *K*_eq_ decreases by ∼6.69 times and *k*_2_ value increases ∼1.26 times with increasing temperature from 275 to 320 K. The rate constant of Channel WM1a (*k*_b_(WM1a)) within the temperature range of 275–320 K is much larger than the corresponding value of *k*_b_(WM1b) in Channel WM1b, given that the ratio of *k*_b_(WM1a)/*k*_b_(WM1b) is 3.67 × 10^3^ to 1.03 × 10^4^. As a result, in the following section, the reaction type where water dimer and water trimer act as a “bridge” will not be considered in the reactions between HO_2_⋯(H_2_O)_2_ + NH_2_ and HO_2_⋯(H_2_O)_3_ + NH_2_.

**Table 1:** Rate constants (cm^3^ per molecule per s) of the HO_2_ + NH_2_ → NH_3_ + ^3^O_2_ reaction with (H_2_O)_*n*_(*n* = 1–3)^a^^,^^b^

*T*(K)	*k*_b_(WM1a)	*k*_b_(WM1b)	*k*_b_(WM2)	*k*_b_(WD1)	*k*_b_(WD2)	*k*_b_(WT1)
275	6.53 × 10^−11^	6.36 × 10^−15^	2.21 × 10^−11^	1.35 × 10^−15^	1.84 × 10^−17^	1.47 × 10^−14^
280	5.23 × 10^−11^	5.85 × 10^−15^	1.70 × 10^−11^	1.39 × 10^−15^	1.92 × 10^−17^	1.45 × 10^−14^
290	3.46 × 10^−11^	5.00 × 10^−15^	1.04 × 10^−11^	1.48 × 10^−15^	2.07 × 10^−17^	1.42 × 10^−14^
298	2.54 × 10^−11^	4.44 × 10^−15^	7.19 × 10^−12^	1.56 × 10^−15^	2.20 × 10^−17^	1.41 × 10^−14^
300	2.38 × 10^−11^	4.32 × 10^−15^	6.62 × 10^−12^	1.58 × 10^−15^	2.23 × 10^−17^	1.40 × 10^−14^
310	1.68 × 10^−11^	3.79 × 10^−15^	4.34 × 10^−12^	1.68 × 10^−15^	2.39 × 10^−17^	1.39 × 10^−14^
320	1.23 × 10^−11^	3.35 × 10^−15^	2.92 × 10^−12^	1.78 × 10^−15^	2.55 × 10^−17^	1.38 × 10^−14^

a *k*_b_(WM1a), *k*_b_(WM1b), *k*_b_(WM2) and *k*_b_(WD1), *k*_b_(WD2), *k*_b_(WT1) is the rate constants of (H_2_O)_*n*_ (*n* = 1–3)-assisted HO_2_ + NH_2_ → NH_3_ + ^3^O_2_ reaction occurring through Channels WM1a, WM1b, WM2 WD1, WD2, and WT1, respectively.

b 1/*k*_uni_(WM1a) = 1/*k*(TS_WM1) + 1/*k*(TS_WM2); 1/*k*_uni_(WM1b) = 1/*k*(TS_WM3) + 1/*k*(TS_WM4); 1/*k*_uni_(WD1) = 1/*k*(TS_WD1) + 1/*k*(TS_WD2); 1/*k*_uni_(WT1) = 1/*k*(TS_WT1) + 1/*k*(TS_WT2).

Differently from Channel WM1a and Channel WM1b above which involve a stepwise process, HO_2_⋯H_2_O + NH_2_ reaction (Channel WM2) contains a one-step mechanism. As for Channel WM2, the reaction started with the formation of a pre-reactive hydrogen bond complex IM_WM5. Compared with the naked complex IM (Fig. 1), in view of geometry, complex IM_WM5 was stabilized by an additional weak hydrogen bond (O3⋯H1, 2.02 Å) and thus, the binding energy of IM_WM5 was enhanced by 1.6 kcal mol^−1^ than that of IM. Starting from IM_WM5, Channel WM2 proceeded through transition state TS_WM5, which is similar in structure to the naked transition state TS, where the N atom of NH_2_ radical directly abstracted the hydrogen of HO_2_. The rate constant of Channel WM2 (*k*_b_(WM2)) listed in Table 1 is only lower by 3–4 times than the corresponding value of *k*_b_(WM1a) in Channel WM1a. However, for H_2_O⋯HO_2_ complex, both the concentration and equilibrium constant at 298 K is larger by 2 orders of magnitude than that of HO_2_⋯H_2_O. As a result, atmospheric relevance of H_2_O⋯HO_2_ + NH_2_ reaction occurring through directing HA will be obvious than that of HO_2_⋯H_2_O + NH_2_ reaction. Besides these, compared with the rate constant of H_2_N⋯H_2_O + HO_2_ reaction (*k*_b_(WM3)) listed in Table S7,† though the value of *k*_b_(WM1a) is smaller by 2 orders of magnitude, atmospheric relevance of H_2_N⋯H_2_O + HO_2_ reaction will be neglected with respect to H_2_O⋯HO_2_ + NH_2_ reaction, due to the fact that the concentration of H_2_N⋯H_2_O complex is much lower than that of H_2_O⋯HO_2_. This result can be further proved by the effective rate constant in the following section. So, atmospheric relevance of H_2_O⋯HO_2_ + NH_2_ reaction occurring through directing HA will be most obvious in water monomer catalyzed HO_2_ + NH_2_ → NH_3_ + ^3^O_2_ reaction.

### 3.4 Potential energy surfaces and the rate constants for HO_2_ + NH_2_ → NH_3_ + ^3^O_2_ reaction with (H_2_O)_2_

**Fig. 4:**
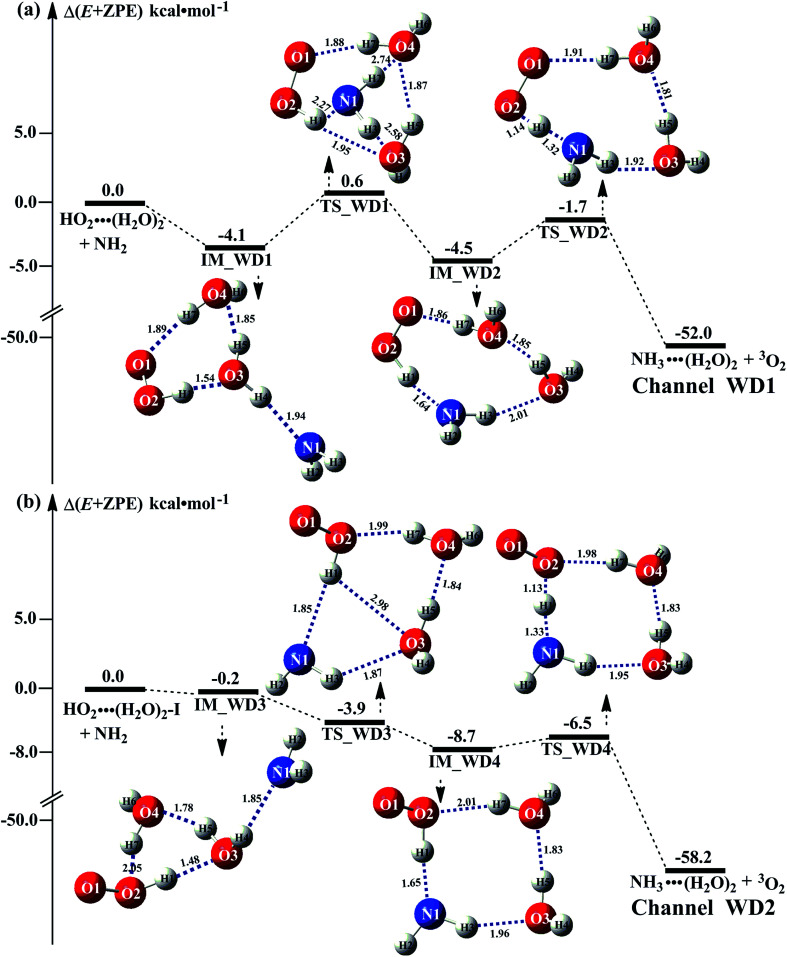
Schematic energy diagrams for water dimer-assisted HO_2_ + NH_2_ → NH_3_ + ^3^O_2_ reaction occurring through HO_2_⋯(H_2_O)_2_ + NH_2_ (a) and HO_2_⋯(H_2_O)_2_–I + NH_2_ (b) reactions; energies (kcal mol^−1^) at the CCSD(T)/CBS//M06-2X/6-311+G(3*df*,2*pd*) level of theory.

As the discussion in Section 3.2 that the atmospheric relevance of HO_2_⋯(H_2_O)_2_ + NH_2_ reaction is most obvious in water dimer-catalyzed the reaction (3). So, Fig. 4 shows the PESs of HO_2_⋯(H_2_O)_2_ + NH_2_ reaction (Channel WD1) with its rate constant listed in Table 1. For comparison, PESs for HO_2_⋯(H_2_O)_2_–I + NH_2_ reaction (Channel WD2) was displayed in Fig. 4, and its corresponding rate constant was shown in Table 1.

For Channels WD1 and WD2 shown in Fig. 4, both reactions proceeded through a stepwise mechanism, where the reaction firstly occurred *via* a ring enlargement, and then proceed through a direct HA. However, the rate constant of Channel WD2 (*k*_b_(WD2)) listed in Table 1 is much smaller than the corresponding value of *k*_b_(WD1) in Channel WD1, given that the ratio of *k*_b_(WD1)/*k*_b_(WD2) is 69.8–73.4. Meanwhile, the value of *k*_b_(WD1) is larger by 6 orders of magnitude than that of *k*_b_(WD3) (H_2_N⋯(H_2_O)_2_ + HO_2_ reaction). Thus, for the reaction (3) with water dimer, HO_2_⋯(H_2_O)_2_ + NH_2_ reaction (Channel WD1) is of great atmospheric relevance due to its larger rate constant, and only this channel has been mainly taken into account here.

Consistent with the favorable channel of H_2_O⋯HO_2_ + NH_2_ reaction (Channel WM1a) above, as for Channel WD1, with the collision between NH_2_ and HO_2_⋯(H_2_O)_2_, the reaction occurred *via* a stepwise mechanism. In the first step, Channel WD1 went through a ring enlargement from seven-membered ring complex IM_WD1 to nine-membered ring complex IM_WD2 *via* transition state TS_WD1 with an energy barrier of 4.7 kcal mol^−1^. In geometrical point of view, complexes IM_WD1 and IM_WD2 have similar structures as the corresponding complexes of IM_WM1 and IM_WM2, expecting that water monomer was substituted by water dimer. So, in energetic point of view, similar with the fact that the complex IM_WM2 in Channel WM1a (Fig. 3) is more stable than IM_WM1, complex IM_WD2 in Channel WD1 is stable than IM_WD1. However, the stabilization energy of IM_WD2 has been reduced by 2.8 kcal mol^−1^ than that of IM_WM2. In the second step, following IM_WD2 complex, with the N atom of NH_2_ abstracting the H atom of HO_2_ moiety in HO_2_⋯(H_2_O)_2_, Channel WD1 can proceed *via* an elementary reaction of direct HA (TS_WD2) to form the products of NH_3_⋯(H_2_O)_2_ + ^3^O_2_. Similar with complex IM_WD2, TS_WD2 also shows a nine-membered ring structure with water dimer, NH_2_ radical and HO_2_ radical involved. The relative energy of TS_WD2 to HO_2_⋯(H_2_O)_2_ + NH_2_ is −1.7 kcal mol^−1^, which is higher by 2.2 kcal mol^−1^ than that of the water-assisted transition state TS_WM2 to H_2_O⋯HO_2_ + NH_2_ reactants. Meanwhile, the rate constant of Channel WD1, as shown in Table 1, is smaller by 3–4 orders of magnitude than that of *k*_b_(WM1a) with single water. Due to the fact that H_2_O⋯HO_2_ has higher concentration than that of HO_2_⋯(H_2_O)_2_, we predict that Channel WD1 has less obvious positive influence on enhancing the rate of the reaction (eqn (3)) than Channels WM1a.

### 3.5 Potential energy surfaces and the rate constants for HO_2_ + NH_2_ → NH_3_ + ^3^O_2_ reaction with (H_2_O)_3_

**Fig. 5:**
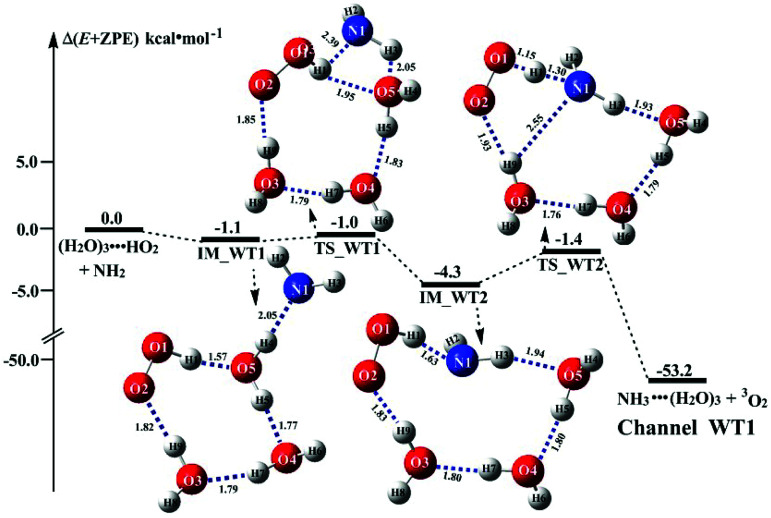
Schematic energy diagrams for water trimer-assisted HO_2_ + NH_2_ → NH_3_ + ^3^O_2_ reaction occurring through HO_2_⋯(H_2_O)_3_ + NH_2_(a) reactions; energies (kcal mol^−1^) at the CCSD(T)/CBS//M06-2X/6-311+G(3*df*,2*pd*) level of theory. ^x^Value was from ref. and key interatomic distances (angstroms) and angles (degrees) of molecular structures was given at the M06-2X/aug-*cc*-pVTZ. ^y^Value was from ref. and key interatomic distances (angstroms) and angles (degrees) of molecular structures was given at the MP2/6-31G* level of theory.

It is of interest to known whether (H_2_O)_3_ will affect the HO_2_ + NH_2_ → NH_3_ + ^3^O_2_ reaction. Thus, based on the discussed results above that the reaction of NH_2_ radical with HO_2_⋯(H_2_O)_2_ is more favorable than the reactions of NH_2_ radical with HO_2_⋯(H_2_O)_2_–I in the presence of water dimer, only the reaction of NH_2_ radical with HO_2_⋯(H_2_O)_3_ was mainly investigated for the channel of NH_3_ + ^3^O_2_ formations with water trimer due to that the binding energy (Table S2†) and the concentration (Table S3†) of HO_2_⋯(H_2_O)_3_ are much larger than those of H_2_N⋯(H_2_O)_3_, HO_2_⋯(H_2_O)_3_–I. The schematic potential energy surfaces for HO_2_⋯(H_2_O)_3_ + NH_2_ reaction was shown in Fig. 5, meanwhile its rate constant is also listed in Table 1, which is larger by 6 orders of magnitude than that of *k*_b_(WT2) (H_2_N⋯(H_2_O)_3_ + HO_2_ reaction) involved in Table S7.†

Regarding the reaction of HO_2_⋯(H_2_O)_3_ + NH_2_, the pre-reactive complex IM_WT1 was formed with the energy of 1.1 kcal mol^−1^ with respect to the HO_2_⋯(H_2_O)_3_ + NH_2_ reactants. Starting from complex IM_WT1, the reaction occurs *via* transition state TS_WT1 and to form a complex IM_WT2 with a barrier of 0.1 kcal mol^−1^ relative to the pre-reactive complex IM_WT1. This step involves a geometric rearrangement that plays a crucial role in HO_2_⋯(H_2_O)_3_ + NH_2_ reaction. IM_WT2 is more stabilized than IM_WT1 by 3.2 kcal mol^−1^. From the geometric point of view, complex IM_WT2 has similar quasi-planar structure as that of IM_WD2 with the additional water molecule inserted between HO_2_ and NH_2_. The relative energy of IM_WT2 is −4.3 kcal mol^−1^ with respect to HO_2_⋯(H_2_O)_3_ + NH_2_.

Transition state TS_WT2 was found between IM_WT2 and the products (H_3_N⋯(H_2_O)_3_ and ^3^O_2_). For the TS_WT2, a HA reaction occurs by the N atom of NH_2_ abstracted the H atom of HO_2_ radical as that in TS_WD2 with the additional water molecule inserted between HO_2_ and NH_2_. TS_WT2 lies 1.4 kcal mol^−1^ below the HO_2_⋯(H_2_O)_3_ + NH_2_ reactants, which is 0.3 kcal mol^−1^ higher in energy than the relative energy of TS_WD2 to HO_2_⋯(H_2_O)_2_ + NH_2_ reactants. Meanwhile, the rate constant of Channel WT1 (shown in Table 1) is larger by 8–11 times than that of *k*_b_(WD1) with water dimer. Due to the fact that HO_2_⋯(H_2_O)_2_ has higher concentration than that of HO_2_⋯(H_2_O)_3_, thus, whether the atmospheric relevance of HO_2_⋯(H_2_O)_3_ + NH_2_ reaction is obvious than that of HO_2_⋯(H_2_O)_3_ + NH_2_ reaction or not, needs further discussion in the next section.

### 3.6 Application in atmospheric chemistry

Beyond above mechanisms and rate constant without and with (H_2_O)_*n*_ (*n* = 1–3), another aim of our work was to study the influence of (H_2_O)_*n*_ (*n* = 1–3) on the HO_2_ + NH_2_ reaction under atmospheric conditions. Thus, it is necessary to compare the title rate in the absence of (H_2_O)_*n*_ (*n* = 1–3) with the effective rates of the favorable reactions in the presence of (H_2_O)_*n*_ (*n* = 1–3). Table 2 lists the calculated effective rate constants for the favorable channels of HO_2_ + NH_2_ → NH_3_ + ^3^O_2_ reaction without and with (H_2_O)_*n*_ (*n* = 1–3). For comparisons, the effective rate constants of other channels have been displayed in Table S7.†

**Table 2:** Effective rate constants (cm^3^ per molecule per s) for the favorable channels of HO_2_ + NH_2_ → NH_3_ + ^3^O_2_ reaction with (H_2_O)_*n*_ (*n* = 1–3)^a^^,^^b^^,^^c^

*T*(K)	*k*_R_1__	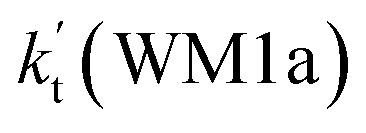	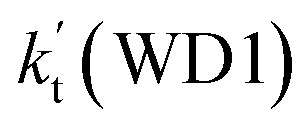	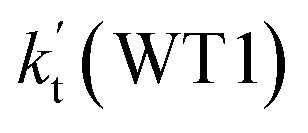	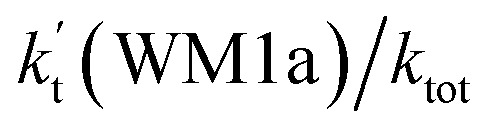
275	5.50 × 10^−11^	6.02 × 10^−12^	1.33 × 10^−18^	1.40 × 10^−19^	10.06%
280	4.72 × 10^−11^	5.25 × 10^−12^	1.51 × 10^−18^	1.69 × 10^−19^	10.24%
290	3.52 × 10^−11^	4.20 × 10^−12^	2.05 × 10^−18^	2.60 × 10^−19^	10.94%
298	2.82 × 10^−11^	3.59 × 10^−12^	2.64 × 10^−18^	3.76 × 10^−19^	11.64%
300	2.68 × 10^−11^	3.36 × 10^−12^	2.77 × 10^−18^	4.05 × 10^−19^	11.47%
310	2.08 × 10^−11^	2.90 × 10^−12^	3.59 × 10^−18^	5.90 × 10^−19^	12.64%
320	1.64 × 10^−11^	2.42 × 10^−12^	4.43 × 10^−18^	7.99 × 10^−19^	13.30%

a Effective rate constants (cm^3^ per molecule per s) of 
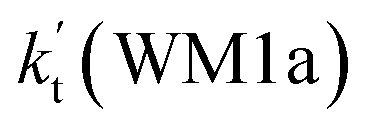
 and 
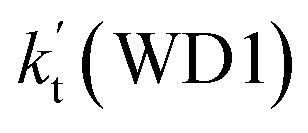
, and 
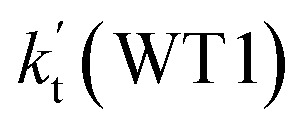
 have been calculated using 100% relative humidity.

b
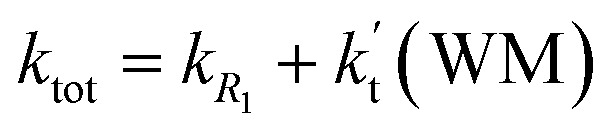
, 
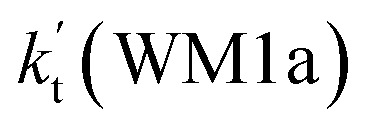
, 
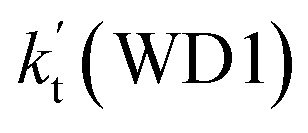
 and 
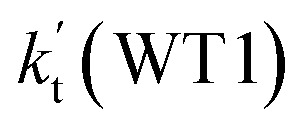
 is respectively the effective rate constants of H_2_O⋯HO_2_ + NH_2_(a), HO_2_⋯(H_2_O)_2_ + NH_2_ and HO_2_⋯(H_2_O)_3_ + NH_2_ reaction.

c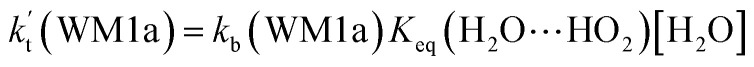; 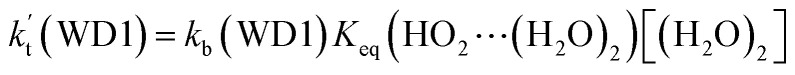; and 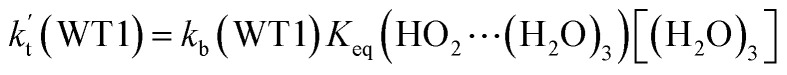. *K*_eq_(H_2_O⋯HO_2_), *K*_eq_(HO_2_⋯(H_2_O)_2_) and *K*_eq_(HO_2_⋯(H_2_O)_3_) is respectively the equilibrium constants for the formation of the H_2_O⋯HO_2_, HO_2_⋯(H_2_O)_2_ and HO_2_⋯(H_2_O)_3_ complexes, whereas [H_2_O], [(H_2_O)_2_], and [(H_2_O)_3_] are the concentrations of water vapor, water dimer and water trimer.^65^

As shown in Table 2, within the temperature range of 275–320 K, the effective rate constant of H_2_O⋯HO_2_ + NH_2_ reaction 
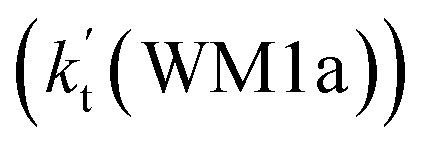
 is 2.42 × 10^−12^ to 6.02 × 10^−12^ cm^3^ per molecule per s, which is larger by 3 orders of magnitude than the corresponding value of HO_2_⋯H_2_O + NH_2_ reaction 
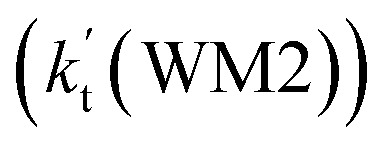
, as shown in Table S7.† Meanwhile, the value of 
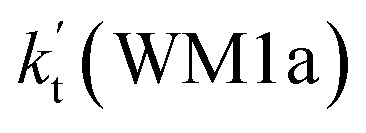
 is respectively larger by 23.5–46.0 and 1.17 × 10^10^ to 4.10 × 10^11^ times than the value of 
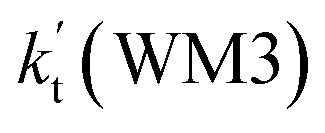
 (H_2_O⋯H_2_N + HO_2_ reaction) and 
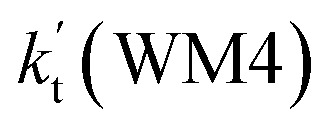
 (H_2_N⋯H_2_O + HO_2_ reaction) listed in Table S7.† This indicates that the catalytic effect of water monomer is mainly taken from H_2_O⋯HO_2_ + NH_2_ reaction.

For the catalytic effect of water dimer, the effective rate constant of 
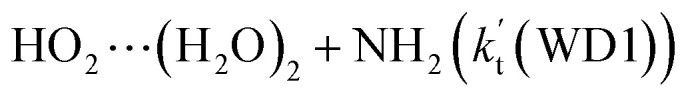
 is 1.33 × 10^−18^ to 4.43 × 10^−18^ cm^3^ per molecule per s, which is larger by 3–4 and 9–10 orders of magnitude than the corresponding value of HO_2_⋯(H_2_O)_2_–I + NH_2_ reaction 
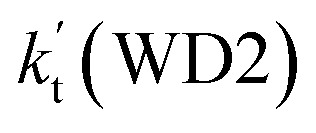
 and 
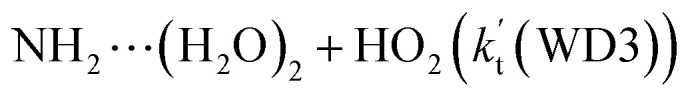
 listed in Table S7,† showing that the catalytic effect of water dimer is mainly taken from HO_2_⋯(H_2_O)_2_ + NH_2_ reaction. Similarity, the catalytic effect of water trimer is mainly taken from HO_2_⋯(H_2_O)_3_ + NH_2_ reaction 
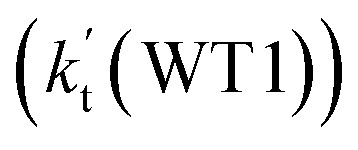
, with the rate constant of 
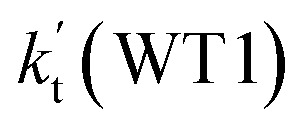
 is larger by 10–11 orders of magnitude than the corresponding value of 
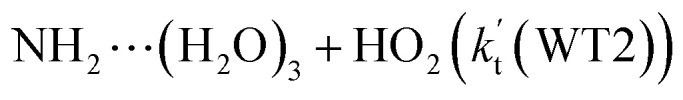
 listed in Table S7.† Besides, the effective rate constant of 
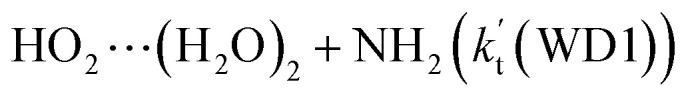
 is larger by 6–10 times than the corresponding value of 
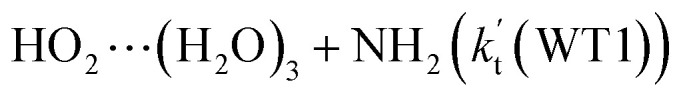
. This shows that compared with water trimer, the catalytic effect of water dimer is not neglected. However, the value of 
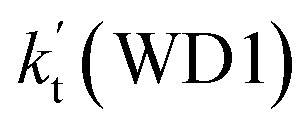
 is smaller by 5–6 orders of magnitude than the effective rate constant of H_2_O⋯HO_2_ + NH_2_ reaction 
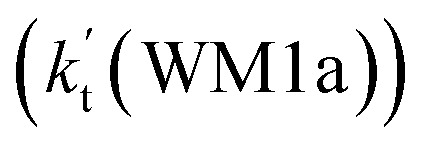
. So, the catalytic effect of single water is the largest among the effect of water, water dimer and water trimer, and the catalytic effect taken from water dimer and water trimer is neglected.

To obtain a more complete understanding of the influence of a water vapor on the title reaction, it is also necessary to compare the title rate constant (*k*_R_1__) in the absence of a water vapor with the effective rate constant of the most favorable channel of H_2_O⋯HO_2_ + NH_2_ reaction 
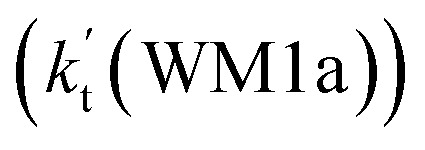
. The result in Table 2 is also estimated that within the temperature range of 275–320 K, the enhancement factor of water vapor 
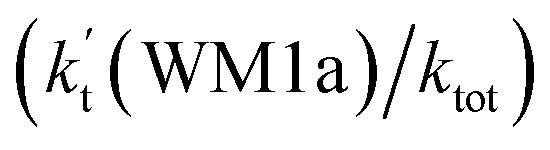
 is 10.06–13.30%, showing, in the whole calculated range, the positive water effect is obvious under atmospheric conditions.

## 4. Summary and conclusions

The HO_2_ + NH_2_ → NH_3_ + ^3^O_2_ reaction catalyzed by (H_2_O)_*n*_ (*n* = 1–3) has been studied theoretically using quantum chemical methods and the canonical variational transition state theory, which results in the following conclusions:

(a) Regarding to each type equilibrium structure of HO_2_⋯(H_2_O)_*n*_ (*n* = 1–3) and H_2_N⋯(H_2_O)_*n*_ (*n* = 1–3), complexes of H_2_O⋯HO_2_, HO_2_⋯H_2_O, HO_2_⋯(H_2_O)_2_, and HO_2_⋯(H_2_O)_3_ are the most stable configurations, which have larger stabilization energies and higher concentrations than their isomers, and thus (H_2_O)_*n*_ (*n* = 1–3) catalyzed HO_2_ + NH_2_ → NH_3_ + ^3^O_2_ reactions are mainly occurring through four kinds of reactions of H_2_O⋯HO_2_ + NH_2_, HO_2_⋯H_2_O + NH_2_, HO_2_⋯(H_2_O)_2_ + NH_2_ and HO_2_⋯(H_2_O)_3_ + NH_2_.

(b) For water-assisted HO_2_ + NH_2_ → NH_3_ + ^3^O_2_ reaction, the channel occurring through the H_2_O⋯HO_2_ + NH_2_ reactants may be of great atmospheric relevance due to its larger effective rate constant and the larger concentration of H_2_O⋯HO_2_. Besides, though HO_2_⋯H_2_O + NH_2_ reaction has lower activation energy, its effective rate constant is smaller by 3 orders of magnitude than the corresponding value of H_2_O⋯HO_2_ + NH_2_ reaction. So, the catalytic effect of water monomer is mainly taken from H_2_O⋯HO_2_ + NH_2_ reaction.

(c) For HO_2_⋯(H_2_O)_2_ + NH_2_ and HO_2_⋯(H_2_O)_3_ + NH_2_ reactions, both the reactions followed through a stepwise mechanism, where the reaction firstly occurred *via* a ring enlargement, and then proceed through a direct HA. However, the effective rate constant of 
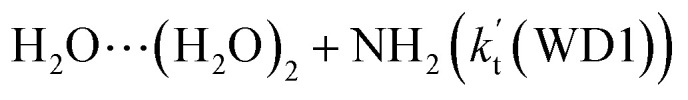
 is larger by 6–10 times than the corresponding value of 
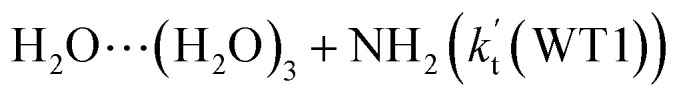
, showing that compared with water trimer, the catalytic effect of water dimer is not neglected. However, the effective rate constant of HO_2_⋯(H_2_O)_2_ + NH_2_ is smaller by 5–6 orders of magnitude than that of H_2_O⋯HO_2_ + NH_2_ reaction, showing that the catalytic effect of single water is the largest among the effect of water, water dimer and water trimer, and the catalytic effect taken from water dimer and water trimer is neglected.

(d) The enhancement factor of water vapor 
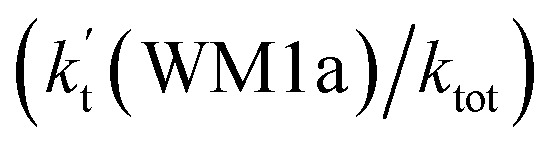
 is 10.06–13.30% within the temperature range of 275–320 K, showing that, in the whole calculated range, the positive water effect is obvious under atmospheric conditions.

## Conflicts of interest

The authors declare no conflicts of interest.

## Supplementary Material

RA-008-C8RA06549G-s001
