# Supplementary material for: Catalytic effect of (H2O)n (n = 1–3) on the HO2 + NH2 → NH3 + 3O2 reaction under tropospheric conditions
Source: RSC Adv. 2018 Nov 5;8(65):37105–16. doi: 10.1039/c8ra06549g (PMC9089316; doi:10.1039/c8ra06549g)
Supplement: RA-008-C8RA06549G-s001 [file RA-008-C8RA06549G-s001.pdf]

Electronic Supplementary Material (ESI) for RSC Advances.
This journal is © The Royal Society of Chemistry 2018

Supporting information

-for-

Catalytic effect of $(H_2O)_n$ (n = 1-3) on the $HO_2 + NH_2 \rightarrow NH_3 + {}^3O_2$ reaction under tropospheric conditions†

Tianlei Zhang*, Kai Wang#, Zhangyu Qiao#, Yongqi Zhang# Lin Geng#, Rui Wang, Zhiyin Wang, Caibin Zhao, Linxia Jin*

Institute of Theoretical and Computational Chemistry, Shaanxi Key Laboratory of Catalysis, School of Chemical & Environment Science, Shaanxi University of Technology, Hanzhong, Shaanxi 723001, China

* Corresponding authors. Tel: +86-0916-2641083, Fax: +86-0916-2641083.
e-mail: ztianlei88@l63.com (T. L Zhang); jinlx@snut.edu.cn (L. X. Jin).
# Kai Wang, Zhangyu Qiao, Yongqi Zhang and Lin Geng contributed equally to this work.

The predicated concentration of NH_2 based on previous experimental reports at 298 K

The main source of NH_2 radical is the reaction of NH_3 with ·OH radical[1]

$$NH_3 + OH \rightarrow H_2O + NH_2 \quad 1.83E\text{-}12 \ (298\ K) \quad k_1 \quad (1)$$

The formed NH_2 is mainly dispelled by the reacts with radicals or molecules, such as NO[2], NO_2[3]

$$NH_2 + NO \rightarrow Products \quad 1.71E\text{-}11 \ (298\ K) \quad k_2 \quad (2)$$

$$NH_2 + NO_2 \rightarrow Products \quad 1.35E\text{-}11 \ (298\ K) \quad k_3 \quad (3)$$

Assumed that the production rate and the depletion rate is comparable, the concentration of NH_2 radical can be approximately equal to:

$$[NH_2] = ([NH_3]\cdot[OH]\cdot k_1)/([NO]\cdot k_2 + [NO_2]\cdot k_3)$$

where k_1, k_2, k_3 are rate constants for the reactions of R1, R2, R3, respectively. It is assumed to be 1ppbv of the OH concentration, while 10ppbv of the concentrations of NO, NO_2, NH_3, So, the concentration of NH_2 radical is calculated to be approximately 6.0×10^{-11} molecules·cm^{-3} in normal atmosphere condition.

Table S1 Rate constants ($cm^3 \cdot molecules^{-1} \cdot s^{-1}$) for main reaction of the $HO_2 + NH_2$ reaction within the temperature range of 275 – 320 K

T(K)	K_{eq}(IM)	k_{TS}	k_{R1}	k_{exp}	Γ_{CVT}^{R1}
275	1.59E-20	3.46E+09	5.50E-11		1.44
280	1.26E-20	3.74E+09	4.72E-11		1.46
290	8.12E-21	4.34E+09	3.52E-11		1.50
298	5.81E-21	4.85E+09	2.82E-11		1.52
300	5.41E-21	4.97E+09	2.68E-11	(7.51E-11)[4] (2.5E-11)[5]	1.53
310	3.70E-21	5.63E+09	2.08E-11		1.57
320	2.60E-21	6.33E+09	1.64E-11		1.60

T(K)	k_{SCT}^{R1}	Γ_{CVT}^{WM1a}	k_{SCT}^{WM1a}	Γ_{CVT}^{WD1}	k_{SCT}^{WD1}
275	3.32	1.38	10.28	21.40	4.86
280	3.14	1.39	9.25	22.09	4.58
290	2.83	1.40	7.60	23.49	4.11
298	2.62	1.41	6.59	24.66	3.80
300	2.58	1.42	6.37	24.96	3.73
310	2.37	1.43	5.43	26.52	3.41
320	2.20	1.44	4.71	28.12	3.14

K_{eq}(IM) is the equilibrium constant for the process of $HO_2 + NH_2 \rightarrow IM$, k_{TS} is the rate constant of the process of $IM \rightarrow TS \rightarrow NH_3 + {}^3O_2$. $k_{R1} = Keq(IM) \cdot k_{TS}$.

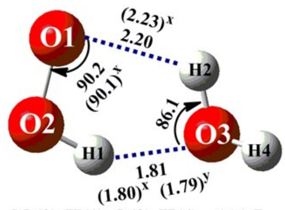

dO(2)-H(1)-O(3)-H(4)= 111.7

$H_2O{\cdots}HO_2(C_1,{}^2A)$

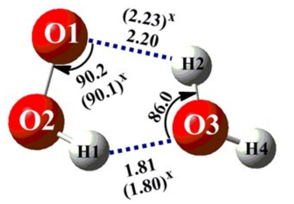

dO(2)-H(1)-O(3)-H(4)= -111.7

$H_2O{\cdots}HO_2a(C_1,{}^2A)$

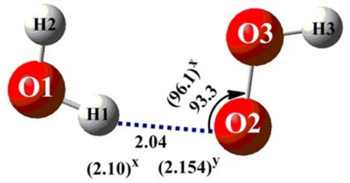

dO(2)-H(1)-O(1)-H(2)= -6.1

$HO_2{\cdots}H_2O(C_1,{}^2A)$

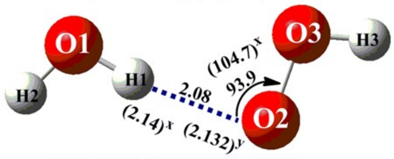

dO(2)-H(1)-O(1)-H(2)= 167.1

$HO_2{\cdots}H_2Oa(C_1,{}^2A)$

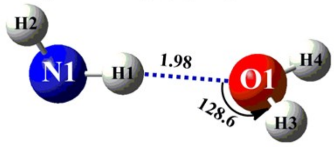

dN(1)-H(1)-O(1)-H(3)= -92.9

$H_2O{\cdots}H_2N(C_1,{}^2A)$

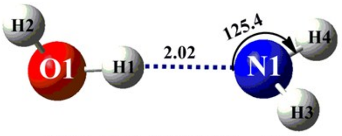

dN(1)-H(1)-O(1)-H(2)= -176.7

$H_2N{\cdots}H_2O(C_1,{}^2A)$

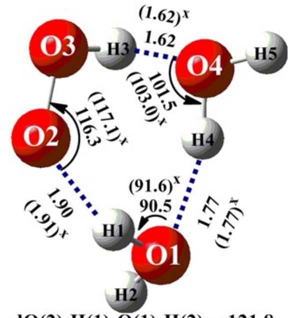

dO(2)-H(1)-O(1)-H(2)= -121.8

$HO_2{\cdots}(H_2O)_2(C_1,{}^2A)$

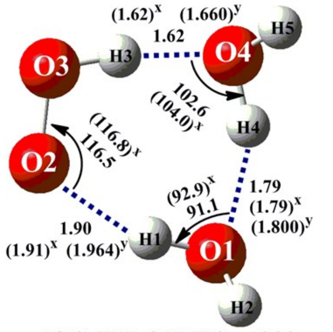

dO(2)-H(1)-O(1)-H(2)= 136.2

$HO_2{\cdots}(H_2O)_2a(C_1,{}^2A)$

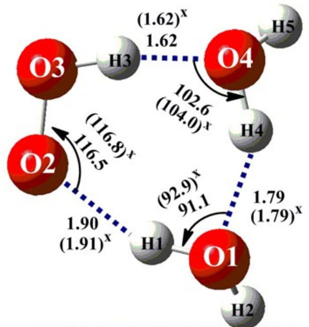

dO(2)-H(1)-O(1)-H(2)= -136.4

$HO_2{\cdots}(H_2O)_2b(C_1,{}^2A)$

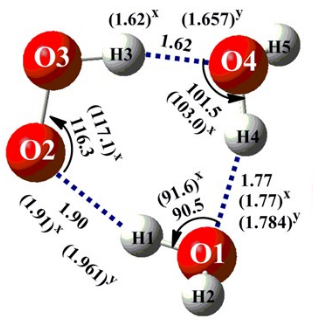

dO(2)-H(5)-O(3)-H(4)= 121.9

$HO_2{\cdots}(H_2O)_2c(C_1,{}^2A)$

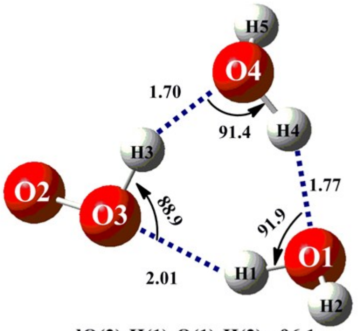

dO(2)-H(1)-O(1)-H(2)= 96.1

$HO_2{\cdots}(H_2O)_2\text{-I}(C_1,{}^2A)$

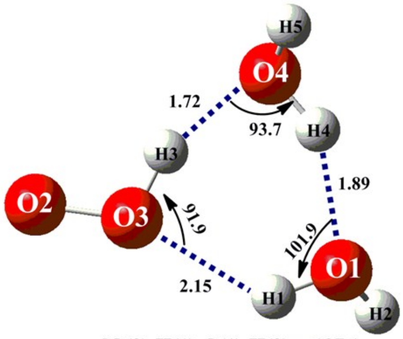

dO(2)-H(1)-O(1)-H(2)= -137.1

$HO_2{\cdots}(H_2O)_2\text{-Ia}(C_1,{}^2A)$

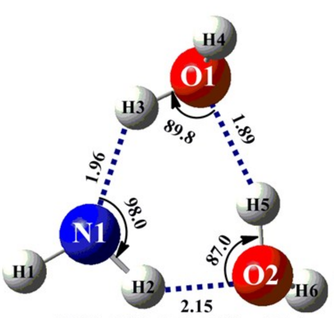

dO(2)-H(5)-O(1)-H(4)= 109.1

$H_2N{\cdots}(H_2O)_2(C_1,{}^2A)$

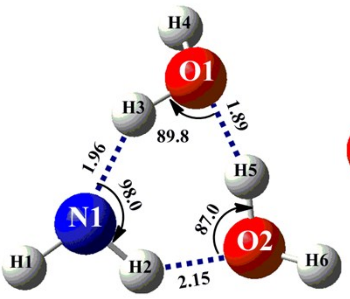

dO(2)-H(5)-O(1)-H(4)= -109.1

$H_2N{\cdots}(H_2O)_2a(C_1,{}^2A)$

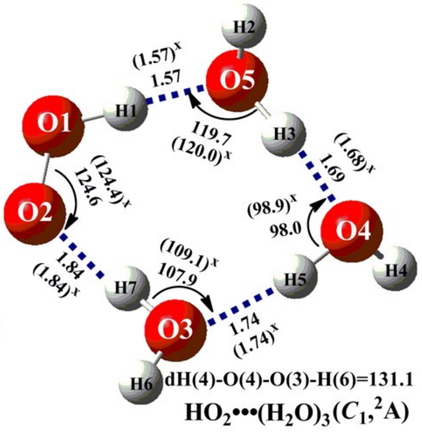

dH(4)-O(4)-O(3)-H(6)=131.1

$HO_2{\cdots}(H_2O)_3(C_1,{}^2A)$

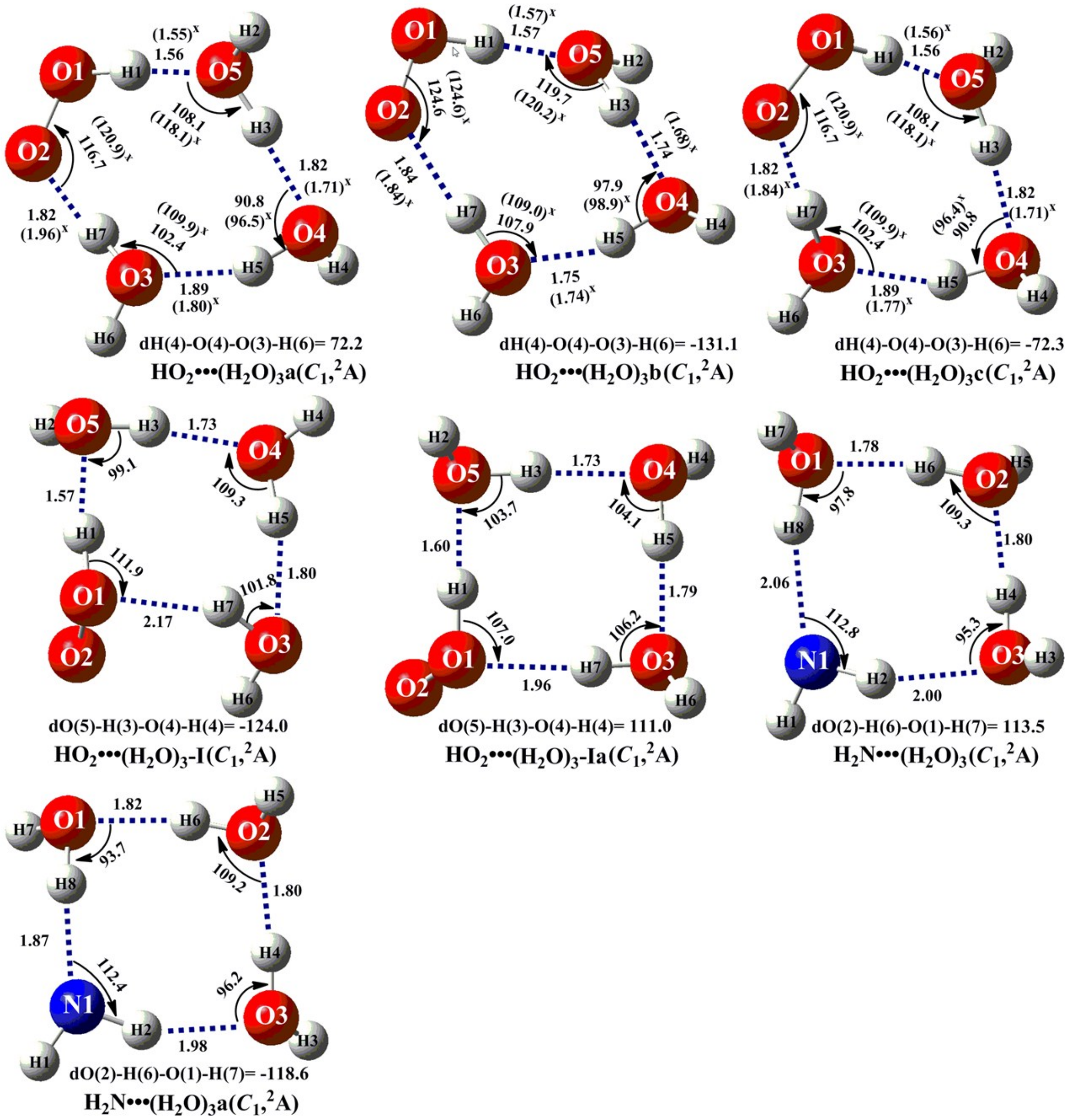

Fig. S1 The geometrical structures of the optimized complexes at the M06-2X/6-311+G(3*df*, 2*pd*) level of theory (bond length Å, bond angle °)$^{x, y}$

^{x} Value was from Ref. [6] and Key interatomic distances (angstroms) and angles (degrees) of molecular structures was given at the M06-2X/aug-cc-pVTZ.

^{y} Value was from Ref. [7] and Key interatomic distances (angstroms) and angles (degrees) of molecular structures was given at the MP2/6-31G* level of theory.

Table S2 Zero point energy (ZPE/(kcal·mol^{-1})), entropies (S/ (cal·mol^{-1}·K^{-1})), relative energies (ΔE and $\Delta(E$ + ZPE)/(kcal·mol^{-1})), enthalpies (ΔH(298)/(kcal·mol^{-1})), and free energies (ΔG(298)/ (kcal·mol^{-1})) for the reactants of $HO_2\cdots(H_2O)_n$ (n = 1-3) and $H_2N\cdots(H_2O)_n$ (n = 1-3)[a]

Species	ZPE	S	ΔE	ΔH	ΔG	$\Delta(E$+ZPE)
$H_2O + HO_2$	22.9	99.6	0.0	0.0	0.0	0.0
$H_2O\cdots HO_2$	25.8	69.9	-9.4	-7.5	1.3	-6.5
$H_2O\cdots HO_2$a	25.8	69.9	-9.4	-7.5	1.3	-6.5
$HO_2\cdots H_2O$	24.8	76.5	-3.3	-1.7	5.2	-1.4
$HO_2\cdots H_2O$a	24.9	75.3	-3.4	-1.8	5.5	-1.4
$H_2O + NH_2$	25.7	91.6	0.0	0.0	0.0	0.0
$H_2O\cdots NH_2$	28.0	69.6	-5.2	-3.4	2.7	-2.9
$NH_2\cdots H_2O$	27.8	70.9	-2.8	-1.3	5.3	-0.6
$H_2O + H_2O$	27.1	90.1	0.0	0.0	0.0	0.0
$(H_2O)_2$	29.3	68.7	-5.2	-3.5	2.9	-2.9
$(H_2O)_2 + HO_2$	38.6	123.3	0.0	0.0	0.0	0.0
$HO_2\cdots(H_2O)_2$	41.9	83.5	-15.8	-13.9	-2.0	-12.6
$HO_2\cdots(H_2O)_2$a	41.9	83.0	-15.4	-13.4	-1.4	-12.1
$HO_2\cdots(H_2O)_2$b	41.9	83.0	-15.4	-13.4	-1.4	-12.1
$HO_2\cdots(H_2O)_2$c	41.8	83.5	-15.8	-13.9	-2.0	-12.6
$HO_2\cdots(H_2O)_2$-I	41.1	88.9	-11.2	-9.4	0.8	-8.7
$HO_2\cdots(H_2O)_2$-Ia	41.1	88.9	-11.0	-9.3	1.0	-8.5
$(H_2O)_2 + NH_2$	41.4	115.2	0.0	0.0	0.0	0.0
$H_2N\cdots(H_2O)_2$	44.4	84.6	-9.1	-7.1	2.1	-6.2
$H_2N\cdots(H_2O)_2$a	44.3	84.6	-9.1	-7.1	2.1	-6.1
$(H_2O)_2 + H_2O$	42.9	113.7	0.0	0.0	0.0	0.0
$(H_2O)_3$	46.1	79.7	-11.1	-9.1	1.0	-7.9
$(H_2O)_3 + HO_2$	55.3	134.3	0.0	0.0	0.0	0.0
$HO_2\cdots(H_2O)_3$	57.8	98.7	-13.8	-12.0	-1.4	-11.5
$HO_2\cdots(H_2O)_3$a	58.2	96.8	-13.5	-11.5	-0.3	-10.7
$HO_2\cdots(H_2O)_3$b	57.8	98.7	-14.0	-12.2	-1.6	-11.5
$HO_2\cdots(H_2O)_3$c	58.2	96.9	-13.6	-11.5	-0.4	-10.7
$HO_2\cdots(H_2O)_3$-I	57.6	100.3	-11.6	-9.9	0.2	-9.5
$HO_2\cdots(H_2O)_3$-Ia	57.5	101.5	-11.7	-9.9	-0.1	-9.4
$NH_2 + (H_2O)_3$	58.1	126.2	0.0	0.0	0.0	0.0
$H_2N\cdots(H_2O)_3$	60.8	97.1	-6.9	-4.8	3.9	-4.2
$H_2N\cdots(H_2O)_3$a	60.8	97.1	-6.7	-4.6	4.1	-4.1

[a] ZPE and S values obtained at M06-2X/6-311+G(3*df*,2*pd*) level of theory; The energy values are obtained at CCSD(T)/CBS level whereas the *H* and *G* corrections are taken from the M06-2X/6-311+G(3*df*,2*pd*) level

Table S3 Equilibrium constants and concentration of $HO_2\bullet\bullet\bullet(H_2O)_n$ (n = 1-3) and $NH_2\bullet\bullet\bullet(H_2O)_n$ (n = 1-3) complexes [a,b,c]

T(K)	$H_2O\cdots HO_2$	$H_2O\cdots HO_2$a	$HO_2\cdots H_2O$	$HO_2\cdots H_2O$a	$H_2O\cdots H_2N$
275	4.88E-19	4.88E-19	3.58E-22	2.77E-22	1.48E-22
280	3.89E-19	3.89E-19	3.44E-22	2.66E-22	1.45E-22
290	2.54E-19	2.54E-19	3.21E-22	2.46E-22	1.38E-22
298	1.83E-19	1.83E-19	3.05E-22	2.33E-22	1.34E-22
298	(4.24E+07) [c]	(4.24E+07) [c]	(7.07E+04) [c]	(5.40E+04) [c]	(6.21E-15) [c]
300	1.71E-19	1.71E-19	3.01E-22	2.30E-22	1.33E-22
310	1.18E-19	1.18E-19	2.85E-22	2.16E-22	1.29E-22
320	8.36E-20	8.36E-20	2.71E-22	2.04E-22	1.25E-22
T(K)	$H_2N\cdots H_2O$	$HO_2\cdots(H_2O)_2$	$HO_2\cdots(H_2O)_2$a	$HO_2\cdots(H_2O)_2$b	$HO_2\cdots(H_2O)_2$c
275	1.25E-20	8.33E-18	2.68E-18	2.71E-18	8.23E-18
280	1.14E-20	5.39E-18	1.76E-18	1.78E-18	5.32E-18
290	9.57E-21	2.36E-18	7.96E-19	8.05E-19	2.33E-18
298	8.40E-21	1.26E-18	4.33E-19	4.38E-19	1.24E-18
298	(3.90E-13) [c]	(5.14E+05) [c]	(1.77E+05) [c]	(1.79E+05) [c]	(5.06E+05) [c]
300	8.16E-21	1.10E-18	3.79E-19	3.83E-19	1.08E-18
310	7.04E-21	5.34E-19	1.90E-19	1.92E-19	5.27E-19
320	6.14E-21	2.72E-19	9.91E-20	1.00E-19	2.69E-19
T(K)	$HO_2\cdots(H_2O)_2$-I	$HO_2\cdots(H_2O)_2$-Ia	$H_2N\cdots(H_2O)_2$	$H_2N\cdots(H_2O)_2$a	$HO_2\cdots(H_2O)_3$
275	3.14E-20	3.14E-20	1.58E-21	1.56E-21	2.90E-18
280	2.35E-20	2.35E-20	1.28E-21	1.26E-21	1.99E-18
290	1.36E-20	1.36E-20	8.55E-22	8.43E-22	9.68E-19
298	8.99E-21	8.99E-21	6.29E-22	6.20E-22	5.59E-19
298	(3.67E+03) [c]	(3.67E+03) [c]	(5.13E-17) [c]	(5.06E-17) [c]	(8.02E+03) [c]
300	8.21E-21	8.21E-21	5.88E-22	5.80E-22	4.95E-19
310	5.12E-21	5.12E-21	4.15E-22	4.09E-22	2.65E-19
320	3.29E-21	3.29E-21	3.00E-22	2.96E-22	1.48E-19
T(K)	$HO_2\cdots(H_2O)_3$a	$HO_2\cdots(H_2O)_3$b	$HO_2\cdots(H_2O)_3$c	$HO_2\cdots(H_2O)_3$-I	$HO_2\cdots(H_2O)_3$-Ia
275	3.80E-19	2.90E-18	3.85E-19	2.31E-19	1.16E-19
280	2.65E-19	1.99E-18	2.69E-19	1.70E-19	8.49E-20
290	1.34E-19	9.68E-19	1.36E-19	9.45E-20	4.75E-20
298	7.98E-20	5.59E-19	8.08E-20	6.04E-20	3.04E-20
298	(1.14E+03) [c]	(8.02E+03) [c]	(1.16E+03) [c]	(8.66E+02) [c]	(4.36E+02) [c]
300	7.12E-20	4.95E-19	7.21E-20	5.48E-20	2.76E-20
310	3.93E-20	2.65E-19	3.98E-20	3.30E-20	1.67E-20
320	2.26E-20	1.48E-19	2.29E-20	2.05E-20	1.04E-20
T(K)	$H_2N\cdots(H_2O)_3$	$H_2N\cdots(H_2O)_3$a	$[H_2O]$[8]	$[(H_2O)_2]$[8]	$[(H_2O)_3]$[8]

275	6.64E-23	1.03E-23	1.89E+17	1.20E+14	3.29E+12
280	5.75E-23	8.89E-24	2.58E+17	2.04E+14	5.85E+12
290	4.39E-23	6.73E-24	4.78E+17	5.91E+14	1.89E+13
298	3.57E-23	5.43E-24	7.73E+17	1.36E+15	4.78E+13
298	(1.02E-19) [c]	(1.56E-20) [c]			
300	3.42E-23	5.19E-24	8.28E+17	1.62E+15	5.82E+13
310	2.71E-23	4.07E-24	1.46E+18	4.06E+15	1.60E+14
320	2.18E-23	3.24E-24	2.35E+18	9.24E+15	3.91E+14

[a] Equilibrium constants in units of $cm^3 \cdot molecule^{-1}$

[b] All equilibrium constants were calculated by using energies computed at CCSD(T)/CBS level and partition functions obtained at M06-2X/6-311+G(3*df*,2*pd*) level about the relevant complexes $H_2O\cdots HO_2$, $H_2O\cdots HO_2a$, $HO_2\cdots H_2O$, $HO_2\cdots H_2Oa$, $H_2O\cdots H_2N$, $H_2N\cdots H_2O$, $HO_2\cdots(H_2O)_2$, $HO_2\cdots(H_2O)_2a$, $HO_2\cdots(H_2O)_2b$, $HO_2\cdots(H_2O)_2c$, $HO_2\cdots(H_2O)_2$-I, $HO_2\cdots(H_2O)_2$-Ia, $H_2N\cdots(H_2O)_2$, $H_2N\cdots(H_2O)_2a$, $HO_2\cdots(H_2O)_3$, $HO_2\cdots(H_2O)_3a$, $HO_2\cdots(H_2O)_3b$, $HO_2\cdots(H_2O)_3c$, $HO_2\cdots(H_2O)_3$-I, $HO_2\cdots(H_2O)_3$-Ia, $H_2N\cdots(H_2O)_3$, $H_2N\cdots(H_2O)_3a$ at 275 – 320 K

[c] The concentration of the corresponding complexes at 298 K

The concentrations of above complexes presented in the Table S3 were theoretical values. They were calculated using the equation as follows:

$$K_{eq}(T) = \sigma \frac{Q_{complex}}{Q_{R1}Q_{R2}} \exp\left(\frac{E_R - E_{complex}}{RT}\right)$$

The various Q values denote the partition functions of the complexes (such as: $HO_2\cdots H_2O$, $HO_2\cdots(H_2O)_2$), $HO_2\cdots(H_2O)_3$, and reactants R, respectively. All partition functions were obtained using the CCSD(T)/CBS//M06-2X/6-311+G(3df,2pd) method. E_R, and $E_{complex}$ stand for the energies of the species of R and complex, respectively, σ is the symmetry factor. The corresponding equation for calculating the concentration is as follows：

$$[(H_2O)_2] = K_{eq}((H_2O)_2)\,[H_2O]^2$$

$$[(H_2O)_3] = K_{eq}((H_2O)_3)\,[H_2O]\,[(H_2O)_2] = K_{eq}((H_2O)_3)\,K_{eq}((H_2O)_2)\,[H_2O]^3$$

$$[H_2O\cdots HO_2]=K_{eq}(H_2O\cdots HO_2)\,[H_2O]\,[HO_2]$$

$$[H_2O\cdots HO_2a]=K_{eq}(H_2O\cdots HO_2a)\,[H_2O]\,[HO_2a]$$

$$[HO_2\cdots H_2O] = K_{eq}(HO_2\cdots H_2O)\,[HO_2]\,[H_2O]$$

$$[HO_2\cdots H_2Oa] = K_{eq}(HO_2\cdots H_2Oa)\,[HO_2]\,[H_2Oa]$$

$$[H_2O\cdots H_2N] = K_{eq}(H_2O\cdots H_2N)\,[H_2O]\,[H_2N]$$

$$[H_2N\cdots H_2O] = K_{eq}(H_2N\cdots H_2O)\,[H_2N]\,[H_2O]$$

$$[HO_2\cdots(H_2O)_2] = K_{eq}(HO_2\cdots(H_2O)_2)\,[HO_2]\,[(H_2O)_2]$$

$$[HO_2\cdots(H_2O)_2a] = K_{eq}(HO_2\cdots(H_2O)_2a)\,[HO_2]\,[(H_2O)_2a]$$

$$[HO_2\cdots(H_2O)_2b] = K_{eq}(HO_2\cdots(H_2O)_2b)\,[HO_2]\,[(H_2O)_2b]$$

$$[HO_2\cdots(H_2O)_2c] = K_{eq}(HO_2\cdots(H_2O)_2c)\ [HO_2]\ [(H_2O)_2c]$$

$$[HO_2\cdots(H_2O)_2\text{-I}] = K_{eq}(HO_2\cdots(H_2O)_2\text{-I})\ [HO_2]\ [(H_2O)_2\text{-I}]$$

$$[HO_2\cdots(H_2O)_2\text{-Ia}] = K_{eq}(HO_2\cdots(H_2O)_2\text{-Ia})\ [HO_2]\ [(H_2O)_2\text{-Ia}]$$

$$[H_2N\cdots(H_2O)_2] = K_{eq}(H_2N\cdots(H_2O)_2)\ [H_2N]\ [(H_2O)_2]$$

$$[H_2N\cdots(H_2O)_2a] = K_{eq}(H_2N\cdots(H_2O)_2a)\ [H_2N]\ [(H_2O)_2a]$$

$$[HO_2\cdots(H_2O)_3] = K_{eq}(HO_2\cdots(H_2O)_3)\ [HO_2]\ [(H_2O)_3]$$

$$[HO_2\cdots(H_2O)_3a] = K_{eq}(HO_2\cdots(H_2O)_3a)\ [HO_2]\ [(H_2O)_3a]$$

$$[HO_2\cdots(H_2O)_3b] = K_{eq}(HO_2\cdots(H_2O)_3b)\ [HO_2]\ [(H_2O)_3b]$$

$$[HO_2\cdots(H_2O)_3c] = K_{eq}(HO_2\cdots(H_2O)_3c)\ [HO_2]\ [(H_2O)_3c]$$

$$[HO_2\cdots(H_2O)_3\text{-I}] = K_{eq}(HO_2\cdots(H_2O)_3\text{-I})\ [HO_2]\ [(H_2O)_3\text{-I}]$$

$$[HO_2\cdots\ (H_2O)_3\text{-Ia}] = K_{eq}(HO_2\cdots(H_2O)_3\text{-Ia})\ [HO_2]\ [(H_2O)_3\text{-Ia}]$$

$$[H_2N\cdots(H_2O)_3] = K_{eq}(H_2N\cdots(H_2O)_3)\ [H_2N]\ [(H_2O)_3]$$

$$[H_2N\cdots\ (H_2O)_3a] = K_{eq}(H_2N\cdots(H_2O)_3a)\ [H_2N]\ [(H_2O)_3a]$$

The equilibrium constant of $H_2O\bullet\bullet\bullet HO_2$ and $HO_2\bullet\bullet\bullet H_2O$ at 298 K is respectively 1.83×10^{-19}, 3.05×10^{-22} $cm^3\bullet molecule^{-1}$, which is close to the reported by Zhang T et al[6]. The only difference is possible due to the fact that different levels of theory used for the partition functions calculation. Taking into account typical tropospheric concentrations of 6.0×10^{-11} $cm^3\bullet molecule^{-1}$ of NH_2 and 3.0×10^8 $cm^3\bullet$ $molecule^{-1}$ of HO_2, it is estimated that the atmospheric concentration of the $H_2O\bullet\bullet\bullet HO_2$ and $HO_2\bullet\bullet\bullet H_2O$ complex is respectively 4.24×10^7 $cm^3\bullet molecule^{-1}$, 7.07×10^4 $cm^3\bullet molecule^{-1}$, respectively. These results are good agreement with the report of Alongi K S et al[7] that the concentration of $H_2O\bullet\bullet\bullet HO_2$ and $HO_2\bullet\bullet\bullet H_2O$ was respectively 6.6×10^7 $cm^3\bullet molecule^{-1}$ and 6.9×10^4 $cm^3\bullet molecule^{-1}$.

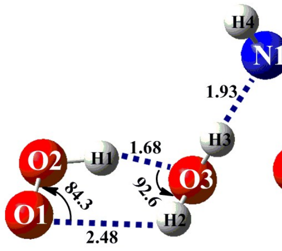

dH(2)-O(3)-H(3)-N(1)= -154.1

IM_WM1 (C_1, ^{3}A)

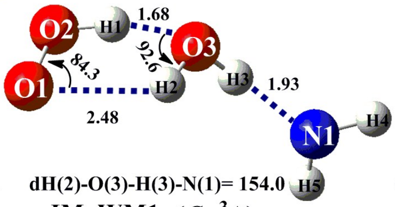

dH(2)-O(3)-H(3)-N(1)= 154.0

IM_WM1a (C_1, ^{3}A)

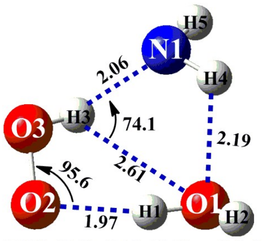

dN(1)-H(4)-O(1)-H(2)= -151.0

TS_WM1 (C_1, ^{3}A)

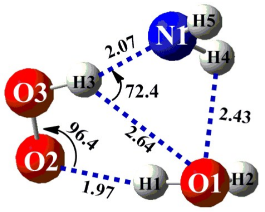

dN(1)-H(4)-O(1)-H(2)= 154.3

TS_WM1a (C_1, ^{3}A)

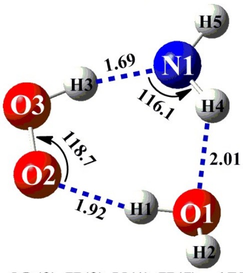

dO(3)-H(3)-N(1)-H(5)= 175.9

IM_WM2 (C_1, ^{3}A)

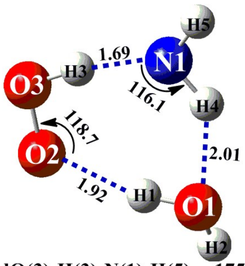

dO(3)-H(3)-N(1)-H(5)= -175.9

IM_WM2a (C_1, ^{3}A)

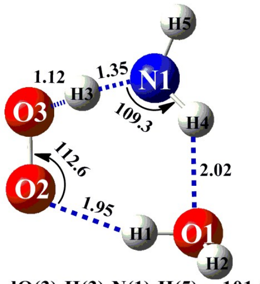

dO(3)-H(3)-N(1)-H(5)= -101.0

TS_WM2 (C_1, ^{3}A)

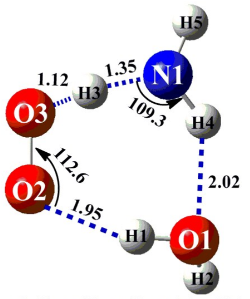

dO(3)-H(3)-N(1)-H(5)= 101.0

TS_WM2a (C_1, ^{3}A)

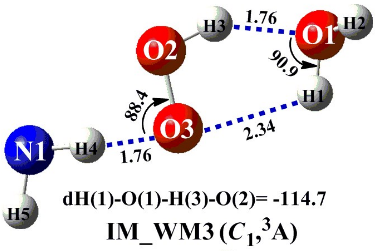

dH(1)-O(1)-H(3)-O(2)= -114.7

IM_WM3 (C_1, ^{3}A)

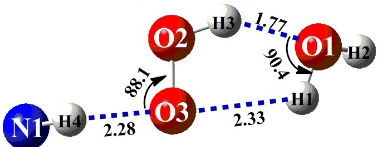

dH(1)-O(1)-H(3)-O(2)= 103.7

IM_WM3a (C_1, ^{3}A)

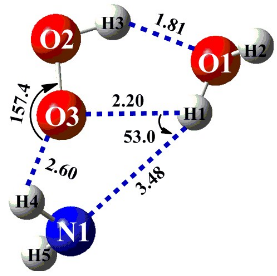

dH(1)-O(1)-H(3)-O(2)= -113.7

TS_WM3 (C_1, ^{3}A)

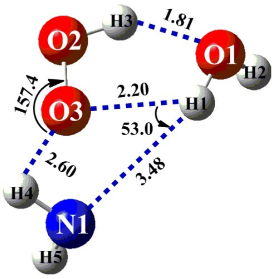

dH(1)-O(1)-H(3)-O(2)= 101.9

TS_WM3a (C_1, ^{3}A)

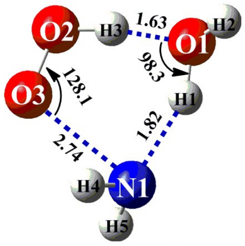

dN(1)-H(1)-O(1)-H(2)= 124.8

IM_WM4 (C_1, ^{3}A)

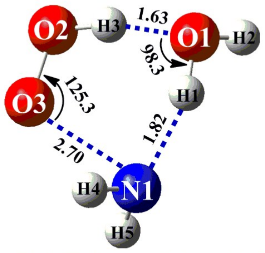

dN(1)-H(1)-O(1)-H(2)= -130.7

IM_WM4a (C_1, ^{3}A)

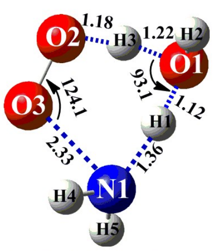

dN(1)-H(4)-O(3)-H(5)= 179.2

TS_WM4 (C_1, ^{3}A)

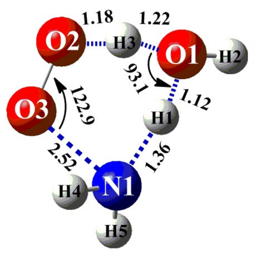

dN(1)-H(1)-O(1)-H(2)= -176.0

TS_WM4a (C_1, ^{3}A)

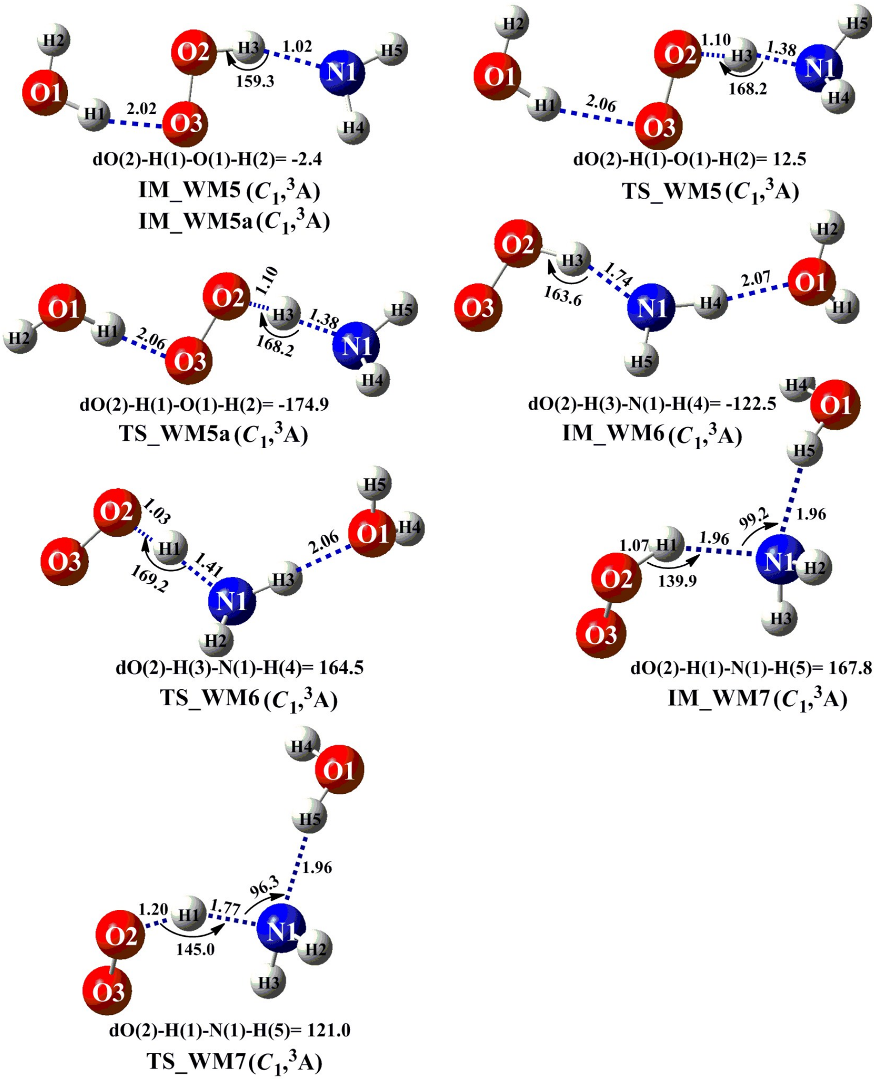

Fig. S2 The geometrical structures of the optimized transitions state, intermediates, and complexes involved in water-assisted $HO_2 + NH_2 \rightarrow NH_3 + {}^3O_2$ reaction

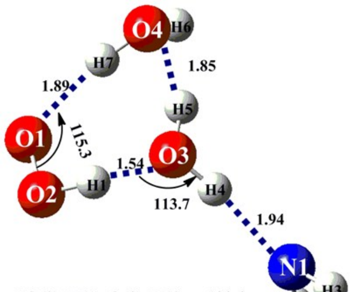

dO(2)-H(1)-O(3)-H(4)= -109.3

IM_WD1(C_1,^{3}A)

IM_WD1a (C_1,^{3}A)

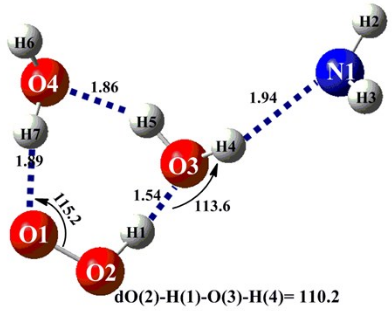

dO(2)-H(1)-O(3)-H(4)= 110.2

IM_WD1b (C_1,^{3}A)

IM_WD1c (C_1,^{3}A)

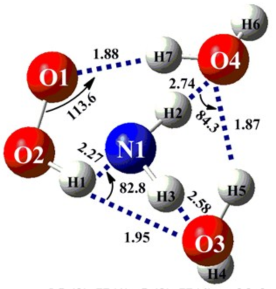

dO(2)-H(1)-O(3)-H(4)= -29.0

TS_WD1(C_1,^{3}A)

TS_WD1a (C_1,^{3}A)

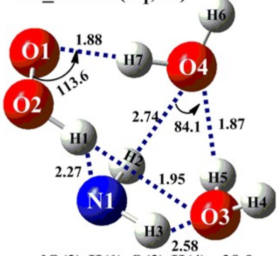

dO(2)-H(1)-O(3)-H(4)= 28.9

TS_WD1b (C_1,^{3}A)

TS_WD1c (C_1,^{3}A)

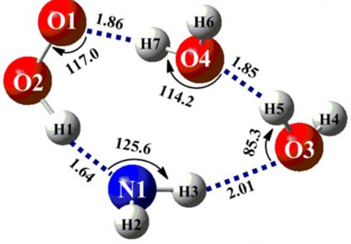

dO(1)-H(7)-O(4)-H(6)= -154.4

IM_WD2 (C_1,^{3}A)

IM_WD2a (C_1,^{3}A)

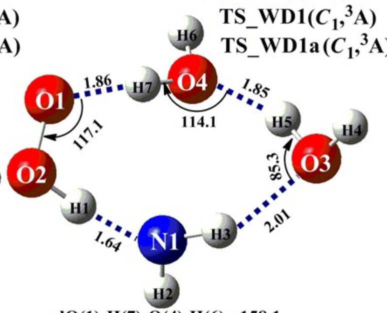

dO(1)-H(7)-O(4)-H(6)= 158.1

IM_WD2b (C_1,^{3}A)

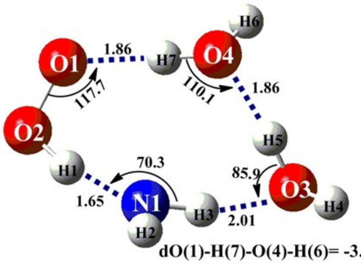

dO(1)-H(7)-O(4)-H(6)= -3.9

IM_WD2c (C_1,^{3}A)

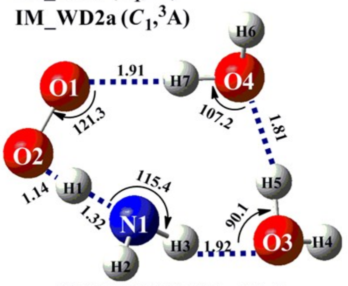

dO(1)-H(7)-O(4)-H(6)= 161.9

TS_WD2 (C_1,^{3}A)

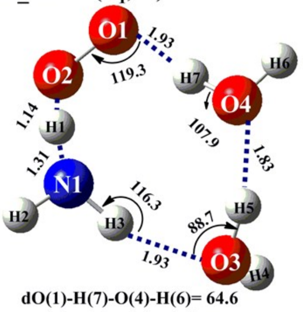

dO(1)-H(7)-O(4)-H(6)= 64.6

TS_WD2a (C_1,^{3}A)

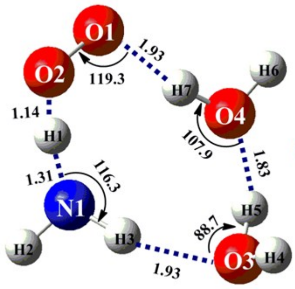

dO(1)-H(7)-O(4)-H(6)= -64.2

TS_WD2b (C_1,^{3}A)

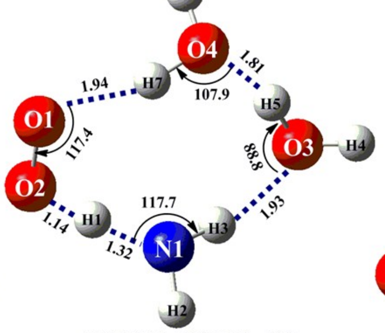

dO(1)-H(7)-O(4)-H(6)= 33.3

TS_WD2c (C_1,^{3}A)

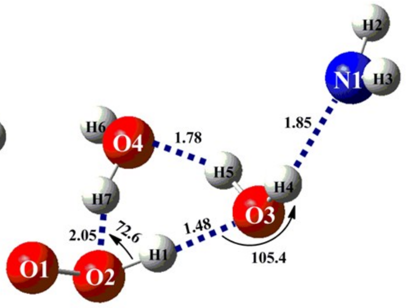

dO(2)-H(1)-O(3)-H(4)= -145.9

IM_WD3 (C_1,^{3}A)

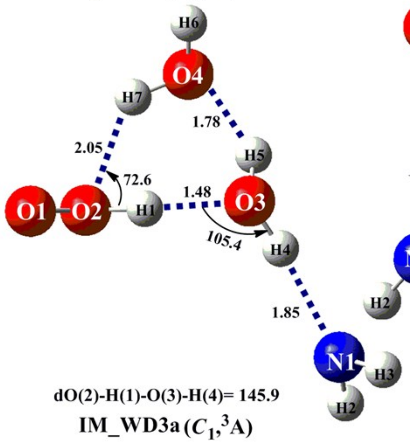

dO(2)-H(1)-O(3)-H(4)= 145.9

IM_WD3a (C_1,^{3}A)

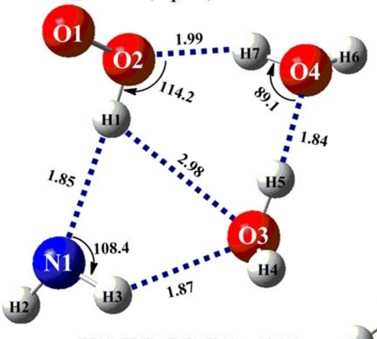

dN(1)-H(3)-O(3)-H(4)= 126.6

TS_WD3 (C_1,^{3}A)

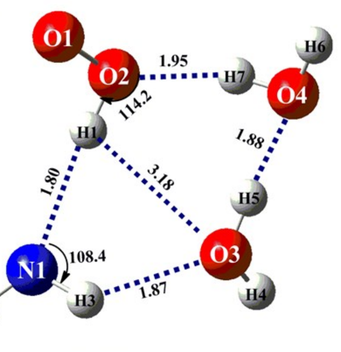

dN(1)-H(3)-O(3)-H(4)= -140.2

TS_WD3a (C_1,^{3}A)

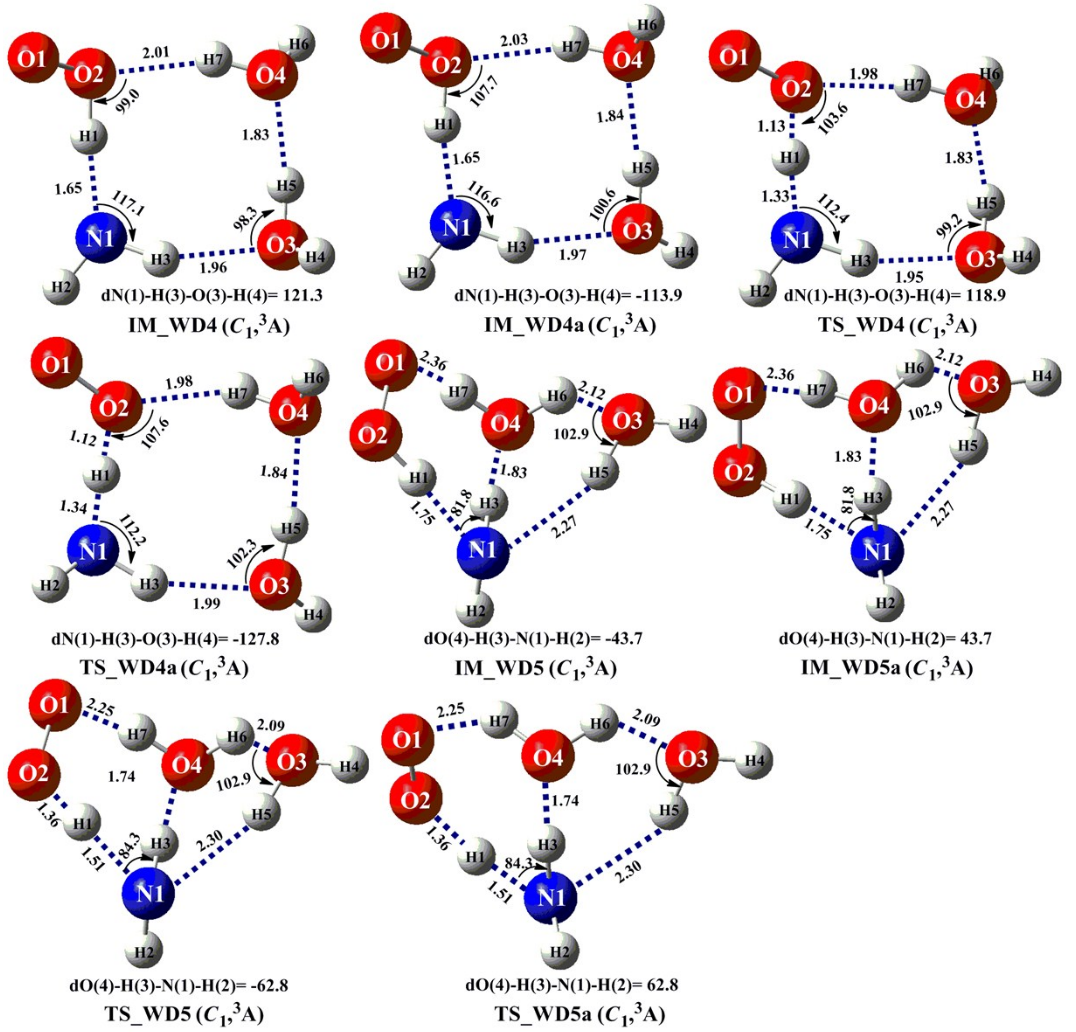

Fig. S3 The geometrical structures of the optimized transitions state, intermediates, and complexes involved in water dimer-assisted $HO_2 + NH_2 \rightarrow NH_3 + {}^3O_2$ reaction

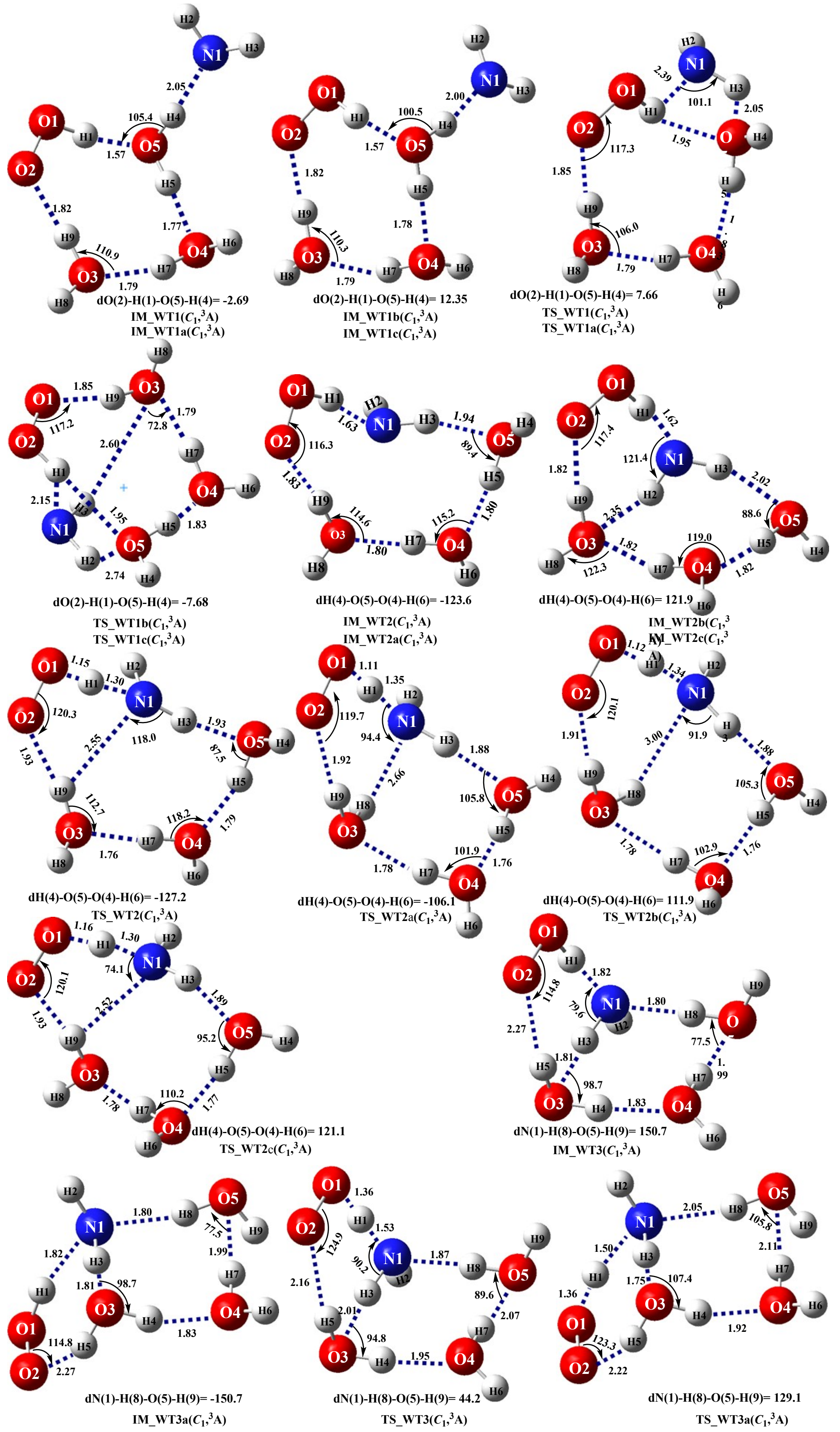

Fig. S4 The geometrical structures of the optimized transitions state, intermediates involved in water trimer-assisted $HO_2 + NH_2 \rightarrow NH_3 + {}^3O_2$ reaction

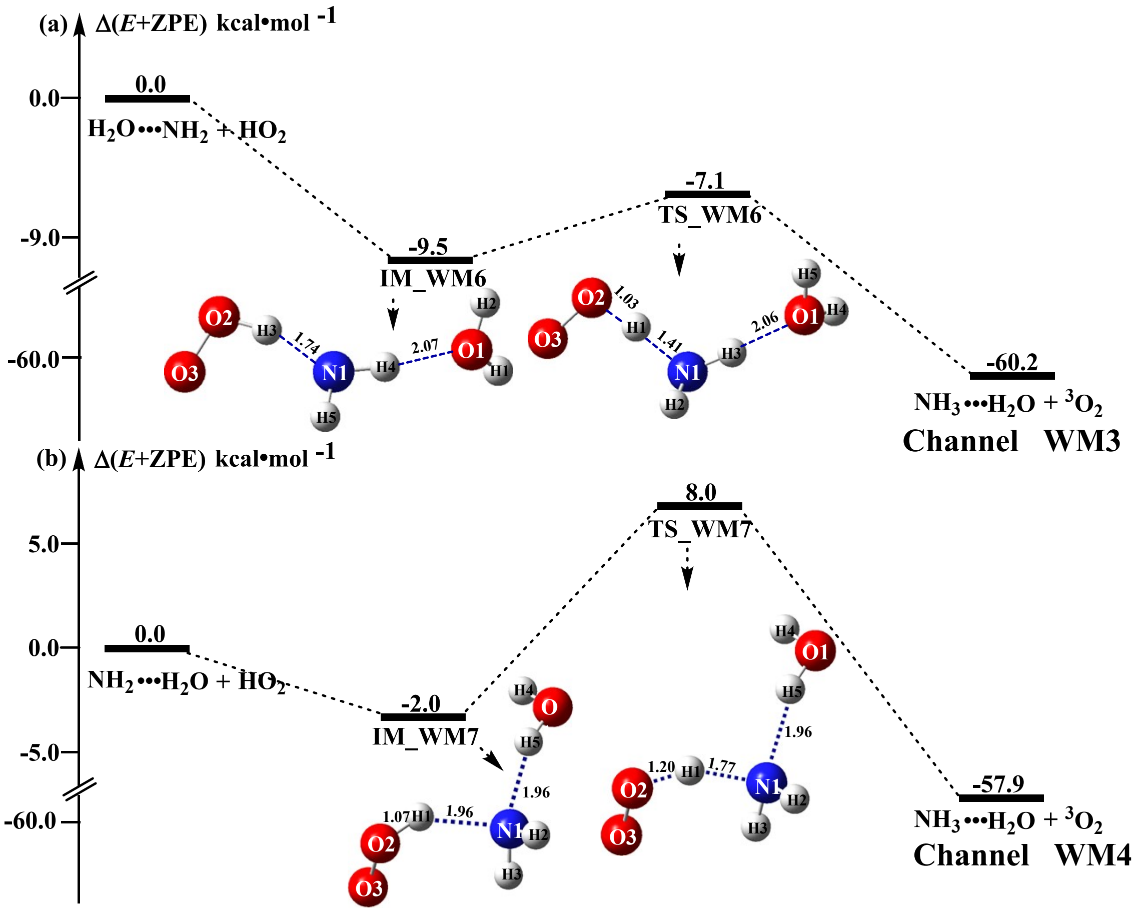

Fig. S5 Schematic energy diagram for the water assisted $HO_2 + NH_2 \rightarrow NH_3 + {}^3O_2$ reaction occurring through $H_2O\cdots H_2N + HO_2$, $H_2N\cdots H_2O + HO_2$ reactions; energies (kcal·mol^{-1}) at the CCSD(T)/CBS//M06-2X/6-311+G(3*df*,2*pd*) level of theory

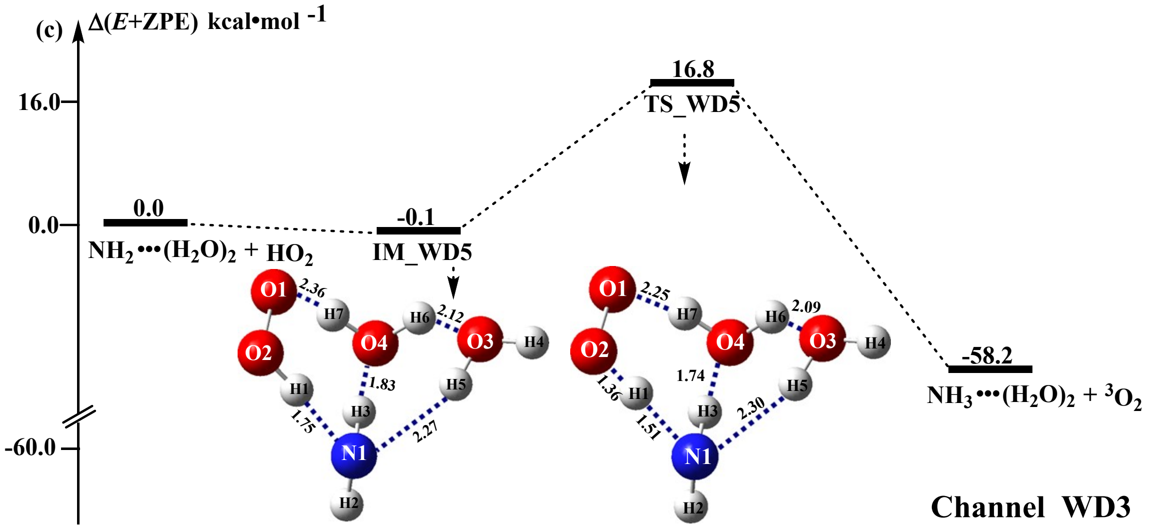

Fig. S6 Schematic energy diagrams for water dimer-assisted $HO_2 + NH_2 \rightarrow NH_3 + {}^3O_2$ reaction occurring through $H_2N\cdots(H_2O)_2 + HO_2$ reaction; energies ($kcal{\cdot}mol^{-1}$) at the CCSD(T)/CBS//M06-2X/6-311+G(3*df*,2*pd*) level of theory

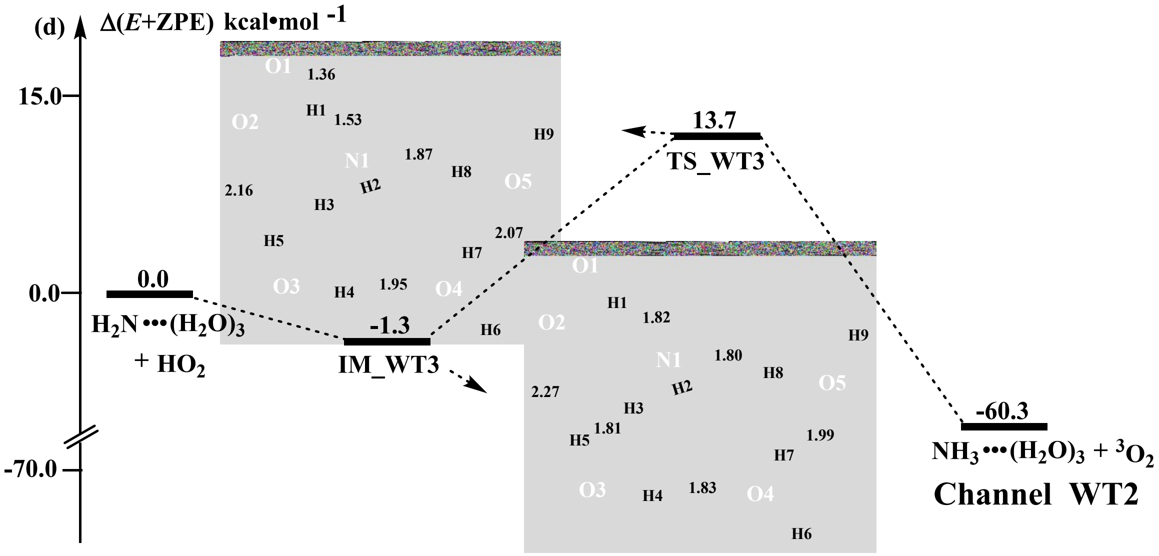

Fig. S7 Schematic energy diagrams for water dimer-assisted $HO_2 + NH_2 \rightarrow NH_3 + {}^3O_2$ reaction occurring through $H_2N\cdots(H_2O)_3 + HO_2$ reaction; energies (kcal·mol^{-1}) at the CCSD(T)/CBS//M06-2X/6-311+G(3*df*,2*pd*) level of theory

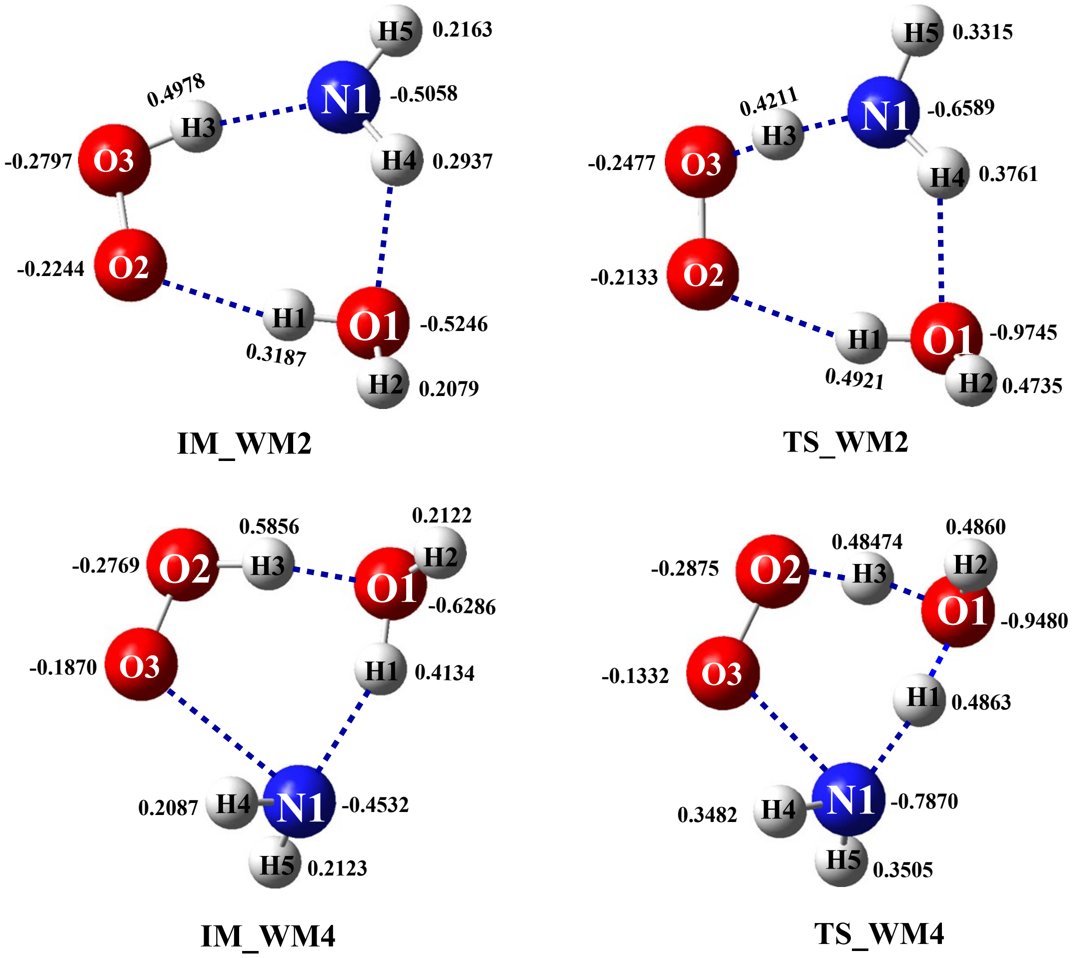

Fig. S8 NBO analysis of charge distribution in IM_WM2 → TS_WM2 and IM_WM4 → TS_WM4

Table S4 Zero-point energy (ZPE/(kcal·mol^{-1})), relative energies (ΔE and $\Delta(E$+ZPE)/(kcal·mol^{-1})), enthalpies (ΔH(298)/(kcal·mol^{-1})), and free energies (ΔG(298)/(kcal·mol^{-1})) for water-assisted the formation of NH_3 + $^3O_2$ from the HO_2 + NH_2 reaction [a]

Species	ZPE	S	ΔE	ΔH	ΔG	$\Delta(E$+ZPE)
$H_2O\cdots HO_2 + NH_2$	37.9	116.4	0.0	0.0	0.0	0.0
$H_2O\cdots NH_2 + HO_2$	37.1	124.1	6.7	6.2	3.9	5.9
IM_WM1	39.9	91.3	-7.5	-5.8	1.7	-5.5
TS_WM1	39.8	83.7	-3.2	-2.3	7.4	-1.2
IM_WM2	40.0	86.8	-9.5	-7.8	1.0	-7.3
TS_WM2	37.2	83.5	-3.3	-4.8	5.0	-3.9
$NH_3\cdots H_2O + {}^3O_2$	40.3	117.5	-56.9	-54.4	-54.7	-54.4
$H_2O\cdots HO_2 + NH_2$	37.9	116.4	0.0	0.0	0.0	0.0
$NH_2\cdots H_2O + HO_2$	37.3	125.5	4.2	4.1	1.4	3.6
IM_WM3	38.4	99.8	-2.6	-1.5	3.5	-2.0
TS_WM3	39.3	86.7	-1.7	-0.9	7.9	-0.2
IM_WM4	40.2	85.3	-8.8	-7.1	2.2	-6.4
TS_WM4	37.9	76.1	1.6	-0.3	11.7	1.6
$NH_3\cdots H_2O + {}^3O_2$	40.3	117.5	-56.9	-54.4	-54.7	-54.4
$HO_2\cdots H_2O + NH_2$	36.8	123.0	0.0	0.0	0.0	0.0
IM_WM5	39.4	93.1	-11.0	-9.1	-0.2	-8.5
TS_WM5	36.4	91.6	-4.8	-6.2	3.1	-5.3
$NH_3\cdots H_2O + {}^3O_2$	40.3	117.5	-63.1	-60.2	-58.6	-59.6
$H_2O\cdots NH_2 + HO_2$	37.1	124.1	0.0	0.0	0.0	0.0
IM_WM6	39.1	95.2	-11.6	-9.7	-1.1	-9.6
TS_WM6	36.3	91.5	-6.4	-7.7	2.0	-7.2
$NH_3\cdots H_2O + {}^3O_2$	40.3	117.5	-63.6	-60.6	-58.6	-60.3
$NH_2\cdots H_2O + HO_2$	37.3	125.5	0.0	0.0	0.0	0.0
IM_WM7	39.1	87.0	-3.6	-3.1	8.4	-1.8
TS_WM7	36.1	84.4	9.4	6.8	19.0	8.2
$NH_3\cdots H_2O + {}^3O_2$	40.3	117.5	-61.2	-58.5	-56.1	-58.1

[a] ZPE values obtained at M06-2X/6-311+G(3*df*,2*pd*) level of theory; The energy values are obtained at CCSD(T)/CBS level whereas the *H* and *G* corrections are taken from the M06-2X/6-311+G(3*df*,2*pd*) level

The method used here for CBS extrapolation was proposed and developed by Varandas and Pansini[9]. In this method the energy at CBS limit has been obtained by extrapolating correlation (Corr) energy and Hatree-Fock (HF) energy separately using two different equations. To extrapolate the correlation (Corr) energy and HF energy following equation was used:

$$CBS(Corr) = \frac{2.71^3 E_{X+1} - 1.91^3 E_X}{2.71^3 - 1.91^3} \quad (1)$$

$$CBS(HF) = \frac{4^5 E_{X+1} - 3^5 E_X}{4^5 - 3^5} \quad (2)$$

Table S5 Zero-point energy (ZPE/(kcal·mol^{-1})), relative energies (Δ*E* and Δ(*E*+ZPE)/(kcal·mol^{-1})), enthalpies (Δ*H*(298)/(kcal·mol^{-1})), and free energies (Δ*G*(298)/(kcal·mol^{-1})) for the HO_2 + NH_2 with two water molecule reaction [a]

Species	ZPE	*S*	Δ*E*	Δ*H*	Δ*G*	Δ(*E*+ZPE)
$HO_2\cdots(H_2O)_2 + NH_2$	53.9	130.0	0.0	0.0	0.0	0.0
IM_WD1	56.2	102.5	-6.4	-4.6	3.6	-4.1
TS_WD1	55.5	98.0	-1.0	0.1	9.6	0.6
IM_WD2	56.1	100.7	-6.7	-4.9	3.8	-4.5
TS_WD2	53.3	98.6	-1.1	-2.5	6.9	-1.7
$NH_3\cdots(H_2O)_2 + {}^3O_2$	56.6	130.7	-54.7	-52.1	-52.3	-52.0
$HO_2\cdots(H_2O)_2$-I + NH_2	54.1	127.2	0.0	0.0	0.0	0.0
IM_WD3	56.9	96.4	-3.0	-1.0	8.2	-0.2
TS_WD3	56.2	95.6	-6.1	-4.8	4.6	-3.9
IM_WD4	55.6	103.8	-10.2	-8.4	-1.4	-8.7
TS_WD4	52.7	102.8	-5.1	-6.5	0.8	-6.5
$NH_3\cdots(H_2O)_2 + {}^3O_2$	56.7	130.7	-60.8	-57.9	-58.9	-58.2
$H_2N\cdots(H_2O)_2 + HO_2$	53.6	139.1	0.0	0.0	0.0	0.0
IM_WD5	55.5	102.5	-2.0	-1.1	9.8	-0.1
TS_WD5	52.2	96.9	18.2	15.5	28.1	16.8
$NH_3\cdots(H_2O)_2 + {}^3O_2$	56.7	130.7	-61.3	-58.7	-56.2	-58.2

[a] ZPE and S values obtained at M06-2X/6-311+G(3*df*,2*pd*) level of theory; The energy values are obtained at CCSD(T)/CBS level whereas the *H* and *G* corrections are taken from the M06-2X/6-311+G(3*df*,2*pd*) level

Table S6 Zero-point energy (ZPE/(kcal·mol^{-1})), relative energies (Δ*E* and Δ(*E*+ZPE)/(kcal·mol^{-1})), enthalpies (Δ*H*(298)/(kcal·mol^{-1})), and free energies (Δ*G*(298)/(kcal·mol^{-1})) for the HO_2 + NH_2 reaction with water trimer [a]

Species	ZPE	S	Δ*E*	Δ*H*	Δ*G*	Δ(*E*+ZPE)
$HO_2\cdots(H_2O)_3 + NH_2$	69.9	145.2	0.0	0.0	0.0	0.0
IM_WT1	72.5	110.4	-3.8	-2.4	8.0	-1.1
TS_WT1	71.1	113.3	-2.2	-1.3	8.2	-1.0
IM_WT2	71.9	117.1	-6.3	-4.6	3.7	-4.3
TS_WT2	69.2	115.3	-0.8	-2.0	6.9	-1.4
$NH_3\cdots(H_2O)_3 + {}^3O_2$	72.9	144.5	-56.1	-53.4	-53.2	-53.2
$NH_2\cdots(H_2O)_3 + HO_2$	70.0	151.7	0.0	0.0	0.0	0.0
IM_WT3	69.1	112.9	-0.4	0.3	11.8	-1.3
TS_WT3	69.0	108.3	14.8	12.3	25.2	13.7
$NH_3\cdots(H_2O)_3 + {}^3O_2$	72.8	144.5	-63.1	-60.7	-58.6	-60.3

[a] ZPE values obtained at M06-2X/6-311+G(3*df*,2*pd*) level of theory; The energy values are obtained at CCSD(T)/CBS level whereas the *H* and *G* corrections are taken from the M06-2X/6-311+G(3*df*,2*pd*) level

Table S7 Rate constants and effective rate constants ($cm^3 \cdot molecule^{-1} \cdot s^{-1}$) for $NH_2 \cdots (H_2O)_n$ (n = 1-3) + HO_2 reaction within the temperature range of 275 - 320 K in the Earth's atmosphere

T/K	k_b(WM3)	k_b(WM4)	k_b(WD3)	k_b(WT2)	k_t'(WM1b)
275	4.68E-09	6.20E-21	4.62E-22	2.93E-21	5.87E-16
280	3.36E-09	6.77E-21	4.78E-22	3.19E-21	5.88E-16
290	1.80E-09	8.12E-21	5.12E-22	3.74E-21	6.07E-16
298	1.12E-09	9.44E-21	5.43E-22	4.25E-21	6.29E-16
300	1.00E-09	9.78E-21	5.50E-22	4.38E-21	6.12E-16
310	5.84E-10	1.18E-20	5.92E-22	5.10E-21	6.53E-16
320	3.52E-10	1.43E-20	6.39E-22	5.92E-21	6.59E-16
T/K	k_t'(WM2)	k_t'(WM3)	k_t'(WM4)	k_t'(WD2)	k_t'(WD3)
275	1.49E-15	1.31E-13	1.47E-23	6.93E-23	8.67E-29
280	1.51E-15	1.26E-13	1.99E-23	9.19E-23	1.23E-28
290	1.60E-15	1.19E-13	3.71E-23	1.67E-22	2.55E-28
298	1.69E-15	1.16E-13	6.13E-23	2.69E-22	4.58E-28
300	1.65E-15	1.11E-13	6.61E-23	2.97E-22	5.17E-28
310	1.80E-15	1.10E-13	1.22E-22	4.97E-22	9.84E-28
320	1.86E-15	1.03E-13	2.07E-22	7.76E-22	1.75E-27
T/K	k_t'(WT2)	K_{eq}(WM1a)	k_2(WM1a)		
275	6.41E-31	3.73E-20	1.75E+10		
280	1.07E-30	2.93E-20	1.79E+10		
290	3.10E-30	1.85E-20	1.88E+10		
298	7.26E-30	1.30E-20	1.96E+10		
300	8.71E-30	1.20E-20	1.97E+10		
310	2.21E-29	8.08E-21	2.08E+10		
320	5.05E-29	5.58E-21	2.20E+10		

k_b(WM3), k_b(WM4), k_b(WD3) and k_b(WT2) is the rate constants of $(H_2O)_n$ (n = 1-3)-assisted $HO_2 + NH_2 \rightarrow NH_3 + {}^3O_2$ reaction occurring through Channels WM3, WM4, WD3 and WT2, respectively;

k_t'(WM1b), k_t'(WM2), k_t'(WM3), k_t'(WM4), k_t'(WD2), k_t'(WD3), and k_t'(WT2) is the effective rate constants of $(H_2O)_n$ (n = 1-3)-assisted $HO_2 + NH_2 \rightarrow NH_3 + {}^3O_2$ reaction occurring through Channels WM1b, WM2, WM3, WM4, WD2, WD3 and WT2, respectively.

References:

[1] K. Clarke, R. Edge, V. Johnson, E. J. Land, S. Navaratnam and T. G. Truscott, *J. Phys. Chem. A*. 2008, 112, 1234-1237.

[2] S. Song, R. K. Hanson, C. T. Bowman and D. M. Golden, D. M. *Int. J. Chem. Kinet*. 2001, 33, 715-721.

[3] H. Meunier, P. Pagsberg and A. Sillesen, *Chem. Phys. Lett.* 1996, 261, 277-282.

[4] O. M. Sarkisov, S. G. Cheskis, V. A. Nadtochenko, E. A. Sviridenkov and V. I. Vedeneev, *Arch.*

Combust. 1984, 4, 111-120
[5] S. G. Cheskis and O. M. Sarkisov, *Chem. Phys. Lett.* 1979, 62, 72-76.
[6] T. L. Zhang, X. G. Lan, Z. Y. Qiao, R. Wang, X. H. Yu, Q. Xu, Z. Y. Wang, L. X. Jin and Z. Q. Wang, *Phys. Chem. Chem. Phys.* 2018, 20, 8152-8165.
[7] K. S. Alongi, T. S. Dibble, G. C. Shields and K. N. Kirschner, *J. Phys. Chem. A*. 2006, 110, 3686-3691.
[8] J. M. Anglada, G. J. Hoffman, L. V. Slipchenko, M. M. Costa, M. F. Ruiz-López and J. S. Francisco, *J. Phys. Chem. A.* 2013, 117,10381-10396.
[9] A. J. C. Varandas and F. N. N. Pansini, *J. Chem. Phys*. 2014, 141, 224113.
